# Water-Assisted Microwave Processing: Rapid Detoxification and Antioxidant Enhancement in Colored Kidney Beans

**DOI:** 10.3390/foods14203557

**Published:** 2025-10-18

**Authors:** Song Yu, Yutao Zhang, Yifei Zhang, Chunyu Zhang, Xinran Liu, Yingjie Wang, Fandi Meng, Lihe Yu

**Affiliations:** 1 College of Agriculture, Heilongjiang Bayi Agricultural University, Daqing 163319, China; byndys@163.com (S.Y.); 13904567536@163.com (Y.Z.); 13684592065@163.com (C.Z.); lxr12132002@163.com (X.L.); 18245720320@163.com (Y.W.); 18746693380@163.com (F.M.); 2 Heilongjiang Provincial Key Laboratory of Modern Agricultural Cultivation and Crop Germplasm Improvement, Daqing 163319, China; 3 Key Laboratory of Low-Carbon Green Agriculture in Northeastern China, Ministry of Agriculture and Rural Affairs, Daqing 163319, China

**Keywords:** antioxidant enhancement, microwave processing, detoxification, kidney beans, metabolomics

## Abstract

This study aimed to develop an industrially viable method for rapid detoxification of raw kidney beans (*Phaseolus vulgaris* L.) while enhancing nutritional properties. Through optimized 5 min water soaking combined with intermittent microwaving (500 W, 5 min)**,** we achieved significant reductions in key antinutrients, namely phytic acid (43–49%), tannins (74–90%), and saponins (59–68%)—all below safety thresholds—concurrently elevating antioxidant capacity (e.g., Ferric Reducing Antioxidant Power: +66–115%) across four colored varieties. Metabolomic analysis of 412 identified metabolites revealed substantial accumulation of key antioxidants including glutathione and quercetin derivatives. Pathway analysis demonstrated dual mechanisms: (1) Detoxification via activated phenylpropanoid biosynthesis degrading tannin precursors and glutathione metabolism reducing phytate; (2) Nutrient enrichment through upregulated phenolic biosynthesis and color-specific flavonoid/betalain pathways. This integrated approach achieves comparable detoxification to 30 min boiling in just 5 min, establishing water-assisted microwave processing as an efficient strategy for industrial-scale production of safer, nutrient-enhanced legumes.

## 1. Introduction

Kidney bean (*Phaseolus vulgaris* L.) seeds are valued for rich antioxidant nutrients (e.g., phenolics, flavonoids) [1,2]. However, they contain significant antinutritional compounds (e.g., phytate, saponins, phytohemagglutinins) conferring mild toxicity [3,4]. A critical challenge in legume processing is achieving effective detoxification while minimizing the loss of valuable nutrients. While traditional boiling reduces toxins effectively, it causes substantial leaching (>50%) of water-soluble antioxidants, diminishing nutritional benefits [5,6].

Microwave processing offers a rapid alternative, minimizing leaching while potentially enhancing antioxidants [7,8]. However, its effectiveness in degrading specific antinutritional factors (e.g., phytate, tannins) is generally less optimal than boiling. Studies on *dehydrated* seeds suggest that hydration pretreatment before microwaving significantly boosts antioxidant levels [9,10]. Recent studies further highlight the role of hydration in enhancing processing efficiency and nutrient retention; for instance, ultrasonic-assisted soaking was shown to improve hydration kinetics and leach antinutrients in red kidney beans [11], while stepwise microwave drying combined with hot air enhanced quick-cooking properties and preserved quality in red beans [12]. Critically, the microwave application mode significantly influences outcomes. Research indicates that *intermittent* microwave treatment (on/off cycles) is superior to continuous processing for preserving and enhancing bioactive compounds in plant tissues due to reduced thermal degradation of heat-sensitive nutrients (phenolics, flavonoids, vitamins), while still eliciting beneficial stress responses promoting antioxidant and secondary metabolite accumulation [8].

Despite the potential of microwave processing, significant knowledge gaps remain regarding its application for simultaneous detoxification and nutrient enhancement in legumes, particularly when combined with hydration pretreatment: (1) The impact of hydration combined specifically with intermittent microwave treatment on the reduction of key antinutrients (e.g., phytate, tannins) in legumes is unclear. (2) While significant color variations exist among kidney beans (white, yellow, red, black), with darker varieties (red/black) exhibiting higher concentrations of both antioxidant phytochemicals and antinutritional compounds [1,13,14,15], research on the differential responses of these colored variants to combined hydration–intermittent microwave processing is scarce. (3) Existing studies on hydration–microwave approaches primarily focus on non-legume models for boosting flavonoids [8] or preserving antioxidants [9,10]; their efficacy and the underlying biochemical shifts for simultaneous detoxification and antioxidant accumulation across diverse bean color genotypes are poorly understood.

We hypothesize that a short-duration water soaking pretreatment followed by intermittent microwave processing will: (i) effectively reduce key antinutritional compounds (phytate, tannins) to safe levels in colored kidney beans, (ii) significantly enhance their antioxidant capacity and associated bioactive compounds, and (iii) elicit color-dependent responses due to inherent differences in pigment-linked metabolic pathways [16]. Addressing these gaps is crucial for developing efficient processing strategies that ensure safety without compromising the inherent nutritional value of differently pigmented beans.

This study therefore investigates the novel combination of short hydration (5 min) and intermittent microwave treatment for simultaneous detoxification and nutrient enhancement across four distinct kidney bean color genotypes (white, yellow, red, black). Objectives are to: (1) Quantitatively assess the efficacy of this combined treatment in reducing key antinutrients (phytate, tannins) and enhancing antioxidant capacity and associated metabolites; (2) Determine if bean color, reflecting underlying metabolic differences, significantly influences sensitivity to the treatment; (3) Utilize non-targeted metabolomics to identify key metabolic pathways altered by processing-induced detoxification and nutrient accumulation, providing foundational insights for future mechanistic studies. Our findings offer a rapid, industrially scalable approach to optimize legume processing, effectively balancing the dual goals of food safety and maximal nutrient preservation, specifically addressing the gap in understanding color-dependent phytochemical stability during intermittent microwave processing.

## 2. Materials and Methods

### 2.1. Materials and Reagents

Kidney bean seeds of various colors—white (Longyun 19), yellow (Qingyun 6), red (British red), and black (Qingyun 1)—were procured from healthy, disease-free plants harvested in the autumn of 2024 at Jian Sanjiang Farm, Heilongjiang, China. Following dehydration, these seeds demonstrated germination rates exceeding 90%. Analytical-grade chemicals, including tripyridyltriazine (TPTZ), diphenylpicrylhydrazine (DPPH), gallic acid (GA), ammonium acetate, ferric chloride hexahydrate (FeCl_3_·6H_2_O), sulfosalicylic acid, Folin–Ciocalteu phenol reagent, sodium carbonate (anhydrous), vanillin, sulfuric acid (95–98%), glacial acetic acid, ammonium hydroxide (25%), sodium acetate trihydrate, and acetone, were acquired from Sigma-Aldrich, Madrid, Spain. High-Performance Liquid Chromatography (HPLC)-grade methanol and acetonitrile were obtained from ANPEL, Shanghai, China, and ultrapure water (18.2 MΩ·cm) was produced using a Milli-Q system (Millipore, MA, USA). All reagents were subjected to purity verification via HPLC prior to experimental application.

The following equipment was utilized: a microwave oven (model: P70D20TL-D4, Galanz, Foshan, China); a forced-air drying oven (model: DHG-9070A, Jing Hong, Shanghai, China); a high-speed centrifuge (model: 5424 R, Eppendorf, Hamburg, Germany); a vacuum concentrator (model: Vacufuge Plus, Eppendorf, Hamburg, Germany); a UV-Vis spectrophotometer (model: UV-2600, Shimadzu, Kyoto, Japan); an Ultra-High Performance Liquid Chromatography (UHPLC) system (Agilent 1290 Infinity LC, Agilent Technologies, Santa Clara, CA, USA); a quadrupole time-of-flight mass spectrometer (SCIEX TripleTOF 6600, SCIEX, Framingham, MA, USA); and an ACQUITY UPLC BEH Amide column (2.1 × 100 mm, 1.7 µm; Waters, Milford, MA, USA).

### 2.2. Experimental Design

This study investigated four uniformly sized, plump, and disease-free kidney bean seed varieties with distinct colors (white, yellow, red, black) to evaluate the effects of intermittent microwave treatment on anti-nutritional compounds, antioxidant capacity, and metabolic profiles. The initial moisture content of the raw seeds, determined by oven drying (105 °C, 24 h) on representative samples, was 6.5% ± 0.3% (dry basis) across all color varieties. Prior to microwave irradiation, seeds in the combined treatment group were soaked in distilled water at room temperature (25 °C) for 5 min, followed by draining. This brief soaking resulted in significant but incomplete water uptake. Water absorption was measured gravimetrically immediately after draining (blotted dry with filter paper to remove surface moisture) and calculated as: [(Weight after soak—Initial dry weight)/Initial dry weight] × 100%. Absorption values were: White beans: 12.4% ± 0.5%, Yellow beans: 10.4% ± 0.7%, Red beans: 9.5% ± 0.5%, and Black beans: 9.3% ± 0.6%. This rapid initial hydration, though substantially less than full saturation typically achieved during prolonged soaking, provided sufficient water to potentiate the subsequent microwave treatment. The selection of a 5 min water pretreatment aligns with emerging strategies to enhance microwave efficacy in seed processing, as demonstrated in studies on oilseeds and legumes [10]. Microwave processing was performed at 500 W (Galanz Microwave Oven, Foshan, China) using an intermittent protocol (1 min on/1 min off, repeated 5 times, totaling 5 min). Seeds were divided into three treatment groups: (1) Control (untreated), (2) Microwave-only (intermittent microwaving without pre-soaking), and (3) Combined treatment (water soaking + intermittent microwaving). For biochemical analyses (phytic acid, tannins, saponins, FRAP, and DPPH), three independent biological replicates per color-treatment subgroup (4 colors × 3 treatments × 3 replicates = 36 samples) were randomly selected and analyzed. For untargeted metabolomic profiling, which focused specifically on comparing the metabolic state of untreated seeds (Control) versus those subjected to the water-assisted microwave process (Combined treatment), seven independent biological replicates per color-treatment subgroup for these two groups (4 colors × 2 treatments × 7 replicates = 56 samples) were utilized. Biochemical data were analyzed using Duncan’s multiple range test (*p* < 0.05, IBM Statistical Product and Service Solutions (SPSS) Statistics 20), ensuring statistically robust comparisons. This experimental design enabled simultaneous assessment of microwave-induced biochemical changes across all treatments and detailed metabolic reprogramming specifically associated with the combined water-microwave processing, with water pretreatment specifically examined for its synergistic effects. The short 5 min soaking step was selected as a minimal pre-treatment to rapidly introduce sufficient water to enhance microwave efficiency without aiming for full rehydration, aligning with the goal of rapid processing.

### 2.3. Antioxidant Capacity Assay

The Ferric Reducing Antioxidant Power (FRAP) assay was performed according to Benzie and Strain [17] with modifications. The FRAP reagent was freshly prepared by mixing 10 mM TPTZ in 40 mM HCl, 20 mM FeCl_3_·6H_2_O, and 300 mM acetate buffer (pH 3.6) in a 1:1:10 (*v*/*v*/*v*) ratio. Briefly, 100 µL of appropriately diluted seed extract was added to 3 mL of FRAP reagent and incubated at 37 °C for 30 min. Absorbance was measured at 593 nm. A standard curve was generated using gallic acid (0–1000 µM; y = 0.0012x + 0.0328, R^2^ = 0.9993), and results were expressed as mmol gallic acid equivalents (GAE) per kg dry weight (DW).

DPPH scavenging activity was determined as per Brand-Williams et al. [18]. A 0.1 mM DPPH solution in methanol was prepared, and 2 mL was mixed with 100 µL of seed extract. After 30 min of incubation in darkness, absorbance was read at 517 nm. The percentage scavenging was calculated as: % scavenging = [(A_0_ − A_1_)/A_0_] × 100, where A_0_ = absorbance of DPPH control and A_1_ = absorbance of sample. For absolute quantification, a Trolox standard curve (0–200 µM; y = 0.3187x + 4.852, R^2^ = 0.9986) was used, and results were expressed as µmol Trolox equivalents (TE) per g DW.

### 2.4. Spectrophotometric Analysis of Seed Anti-Nutrients

Phytate was quantified using the colorimetric method of Latta and Eskin [19]. Briefly, 0.5 g of ground seeds was extracted with 10 mL of 2.4% HCl for 1 h. After centrifugation (4000× *g*, 10 min), 1 mL of supernatant was mixed with 1 mL of Wade’s reagent (0.03% FeCl_3_·6H_2_O and 0.3% sulfosalicylic acid in distilled water). Absorbance was measured at 500 nm. A standard curve was prepared using phytic acid (0–100 µg/mL; y = 0.0094x + 0.071, R^2^ = 0.9991), and results were expressed as mg/g DW.

Total soluble tannins in kidney bean seeds were quantified indirectly as tannic acid equivalents (TAE) using the colorimetric method adapted from Makkar et al. [20], wherein the Folin–Ciocalteu (FC) reagent (diluted 1:10 *v*/*v* with deionized water) reacts with phenolic groups to form a blue chromophore. For analysis, 100 µL of methanolic seed extract was combined with 500 µL of diluted FC reagent and 400 µL of 7.5% (*w*/*v*) Na_2_CO_3_; the mixture was vortexed, incubated for 30 min at 25 ± 1 °C in darkness, centrifuged (4000× *g*, 5 min), and absorbance measured at 725 nm (Shimadzu UV-2600). Quantification employed a tannic acid standard curve (0, 50, 100, 200, 300, 500 µg/mL) with the calibration equation Absorbance = 0.0011 × [Tannic acid] + 0.0215 (R^2^ = 0.9989), and results were expressed as mg TAE/g DW. Although the FC assay broadly detects reducing phenolics, its application for tannin assessment was validated by Makkar et al. [20], who demonstrated strong correlation (r = 0.96, *p* < 0.01) between FC-determined polyphenols and protein-precipitating tannins in legumes via gravimetric analysis.

Saponins were analyzed via the vanillin–sulfuric acid method [21]. Methanolic extract (100 µL) was mixed with 100 µL of 8% (*w*/*v*) vanillin in ethanol and 1 mL of 72% (*v*/*v*) H_2_SO_4_. After heating at 60 °C for 10 min, absorbance was measured at 544 nm. Ginsenoside Re (0–200 µg/mL; y = 0.0043x + 0.105, R^2^ = 0.9978) served as the standard, and results were reported as mg/g DW.

### 2.5. Metabolite Extraction

Seed tissue samples (~80 mg) were cryogenically frozen in liquid nitrogen and manually pulverized into a fine powder. Metabolite extraction was conducted by adding 1000 µL of a pre-chilled solvent system comprising methanol, acetonitrile, and water (2:2:1, *v*/*v*/*v*) to the homogenized powder. The mixture was centrifuged (14,000× *g*, 20 min, 4 °C), and the supernatant was dried under vacuum. For Liquid Chromatography–Mass Spectrometry (LC-MS) analysis, the residue was reconstituted in 100 µL of acetonitrile/water (1:1, *v*/*v*) and recentrifuged (14,000× *g*, 15 min, 4 °C). The clarified supernatant was injected into the LC–MS instrument, following a procedure adapted from De Vos et al. [22].

### 2.6. LC-MS/MS-Based Untargeted Metabolomics Analysis

An Agilent 1290 Infinity UHPLC system coupled to a SCIEX TripleTOF 6600 mass spectrometer was used for metabolite profiling, with modifications from Zhang et al. [23]. Separation was performed on an ACQUITY UPLC BEH Amide column (2.1 × 100 mm, 1.7 µm) with mobile phase A (aqueous 25 mM ammonium acetate and ammonium hydroxide) and B (acetonitrile). The gradient elution was programmed as: 95% B at 0.5 min, decreased linearly to 65% B over 6.5 min, then to 40% B over 1 min, held for 1 min, returned to 95% B within 0.1 min, and re-equilibrated for 3 min. ESI conditions were set as: Gas1 and Gas2 at 60 psi, Curtain Gas (CUR) 30 psi, ion source temperature 600 °C, and ISVF ± 5500 V. Metabolomic data were acquired in both positive and negative ion modes. Full-scan MS (*m*/*z* 60–1000) and data-dependent MS/MS acquisition (*m*/*z* 25–1000) were performed in high-sensitivity mode.

### 2.7. Data Processing and Statistical Analysis

Raw MS data were converted to mzXML using ProteoWizard MSConvert [24] and processed in eXtensible Computational Mass Spectrometry (XCMS) [25] with the centWave algorithm (Δ*m*/*z* = 10 ppm, peakwidth = c(10, 60)). Retention time alignment and feature grouping were conducted using parameters: bw = 5, mzwid = 0.025, and minfrac = 0.5. Isotopic peaks and adducts were annotated using Collective Analysis of Metabolite Experiments with R and Adduct annotation (CAMERA) [26]. Features detected in over 50% of samples in any group were retained. Putative metabolite identification was based on mass accuracy (<10 ppm) and MS/MS spectra matched against a custom standard library.

Statistical analyses were performed using the ropls package in R. Data were sum-normalized and Pareto-scaled prior to Principal Component Analysis (PCA) and Orthogonal Partial Least Squares–Discriminant Analysis (OPLS-DA) [27]. Model validity was assessed via 7-fold cross-validation and permutation tests [28]. Metabolites with Variable Importance in Projection (VIP) > 1, *p* < 0.05, and |FC| > 1.5 were considered significant [29,30]. Pairwise correlations were evaluated using Pearson’s method [31].

## 3. Results

### 3.1. The Effect of Intermittent Microwave Treatment on Kidney Beans

**Figure 1:**
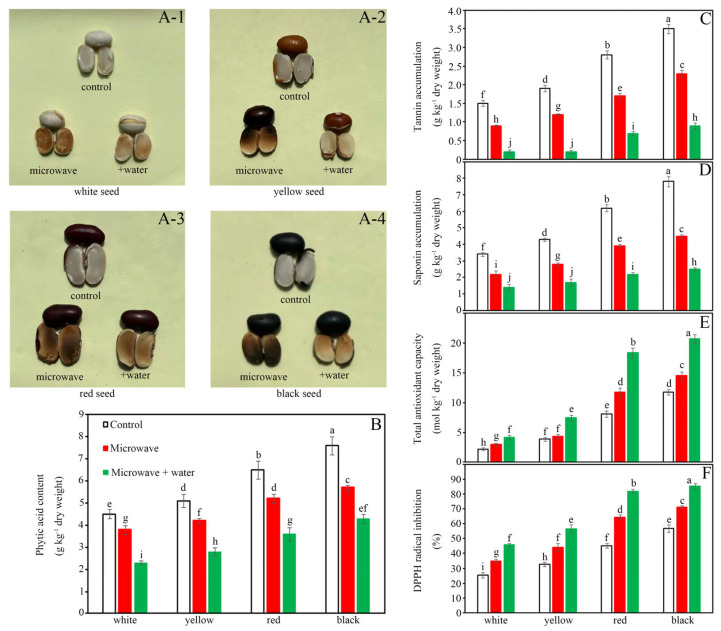
Effects of water soaking pretreatment on antinutritional metabolites and antioxidant nutrient accumulation in four-colored kidney beans under microwave treatment. (**A**) Color changes, (**B**) Phytic acid content, (**C**) Tannin content, (**D**) Saponin content, (**E**) Total antioxidant capacity (FRAP method) and (**F**) DPPH radical scavenging activity. Data are presented as mean ± standard deviation (n = 3). Different lowercase letters above bars within the same variety indicate significant differences (*p* < 0.05) between treatments.

Intermittent microwave treatment (5 min) induced distinct cotyledon discoloration (gray-white to dark/yellowish-brown; Figure 1A), with color-specific severity: black > red > yellow > white beans. Prehydration (5 min) ensured uniform heating and prevented scorching (Figure 1A), maintaining structural integrity across all varieties. Crucially, this brief soaking resulted in significantly different water absorption capacities (*p* < 0.05): white beans absorbed 12.4% ± 0.5% moisture, followed by yellow (10.4% ± 0.7%), red (9.5% ± 0.5%), and black beans (9.3% ± 0.6%). The observed variation in hydration level, particularly the highest in white beans, may contribute to the differential responses in anti-nutrient degradation and antioxidant enhancement following microwave treatment.

Moreover, microwave treatment alone reduced phytic acid content in all four colored beans compared to untreated controls, with decreases of 15.6% (white), 17.6% (yellow), 20.0% (red), and 25.0% (black). The combination of water soaking and microwave treatment further enhanced this reduction, achieving declines of 48.9% (white: 2.3 vs. 4.5 mg/g DW), 45.1% (yellow: 2.8 vs. 5.1 mg/g DW), 44.6% (red: 3.6 vs. 6.5 mg/g DW), and 43.4% (black: 4.3 vs. 7.6 mg/g DW) (Figure 1B). Notably, white beans with the highest water uptake showed the greatest reduction in phytic acid after the combined treatment, suggesting a positive correlation between water absorption and phytic acid degradation. However, the differences among yellow, red, and black beans were marginal, implying that factors beyond hydration may also influence phytate reduction.

Tannin levels were most sensitive to microwave treatments. Direct microwave exposure reduced tannins by 40.0% (white: 0.9 vs. 1.5 mg/g DW), 36.8% (yellow: 1.2 vs. 1.9 mg/g DW), 39.3% (red: 1.7 vs. 2.8 mg/g DW), and 34.3% (black: 2.3 vs. 3.5 mg/g DW). Water soaking prior to microwaving amplified this effect, yielding reductions of 86.7% (white: 0.2 mg/g DW), 89.5% (yellow: 0.2 mg/g DW), 75.0% (red: 0.7 mg/g DW), and 74.3% (black: 0.9 mg/g DW). The synergistic effect highlights the role of water in solubilizing and thermally degrading tannins (Figure 1C). Although yellow beans exhibited the highest tannin reduction after the combined treatment despite intermediate water uptake, the overall high efficiency across all varieties indicates that water facilitates tannin removal, yet seed-specific properties likely modulate the extent of degradation.

Saponin content decreased by 35.3% (white), 34.9% (yellow), 37.1% (red), and 42.3% (black) after microwave treatment alone. Water soaking before microwaving led to more pronounced reductions: 58.8% (white: 1.4 vs. 3.4 mg/g DW), 60.5% (yellow: 1.7 vs. 4.3 mg/g DW), 64.5% (red: 2.2 vs. 6.2 mg/g DW), and 67.9% (black: 2.5 vs. 7.8 mg/g DW). This indicates that saponins, like other anti-nutrients, are susceptible to combined hydothermal and microwave effects (Figure 1D). The greatest saponin reduction was observed in black beans, which had the lowest water absorption, indicating no clear positive correlation between water uptake and saponin degradation. Instead, initial saponin content and seed coat characteristics may play more critical roles.

In contrast to anti-nutrients, total antioxidant capacity (FRAP values) increased with microwave treatment, particularly in water-soaked beans. Direct microwaving elevated FRAP by 32.7% (white: 2.96 vs. 2.23), 12.4% (yellow: 4.35 vs. 3.87), 45% (red: 11.76 vs. 8.11), and 23.7% (black: 14.57 vs. 11.78). Water soaking prior to microwaving further boosted FRAP by 86.5% (white: 4.16), 92.0% (yellow: 7.43), 125.6% (red: 18.34), and 75.5% (black: 20.67), suggesting enhanced release or preservation of antioxidants (Figure 1E). The highest FRAP improvement was noted in red beans after combined treatment, despite their intermediate water absorption, implying that water uptake alone is not the sole determinant of antioxidant enhancement. Pigment-related phytochemicals likely contribute significantly to the total antioxidant capacity.

DPPH scavenging activity improved significantly with both treatments. Microwave exposure alone increased activity by 37.1% (white: 35.1% vs. 25.6%), 35.5% (yellow: 44.3% vs. 32.7%), 42.6% (red: 64.6% vs. 45.3%), and 25.4% (black: 71.2% vs. 56.8%). Water soaking combined with microwaving maximized the effect, achieving 78.9% (white: 45.8%), 73.4% (yellow: 56.7%), 80.8% (red: 81.9%), and 50.5% (black: 85.5%) improvements. This aligns with FRAP data, confirming that water pretreatment optimizes microwave-induced antioxidant activation (Figure 1F). Similar to FRAP, DPPH activity enhancement did not directly correlate with water absorption, further supporting that bean color and associated bioactive compounds are major factors influencing antioxidant properties.

### 3.2. Metabolite Profiling and Statistical Validation of Microwave-Treated Beans

LC-MS/MS analysis in positive and negative ion modes (Table 1 and Table S1) detected 15,816 metabolic features in microwave-treated kidney beans (7374 and 8442 peaks, respectively). Rigorous quality assurance (QA) measures—including randomized sample preparation, solvent blank subtraction, and instrument validation—ensured data integrity. Base peak chromatograms (BPC) showed >88% overlap in QC samples across ion modes, while total ion chromatogram (TIC) overlap exceeded 90%, with retention time deviation < 0.15 min and RSD < 30% for >70% of peaks. Noise filtering (3× baseline intensity) and retention time alignment (<0.05 min tolerance) enhanced signal fidelity, supported by minimal BPC peak shifts (<0.10 min) (Figures S1 and S2). Database matching (HMDB, MassBank, etc.) identified 412 metabolites (Table S2).

**Figure 2:**
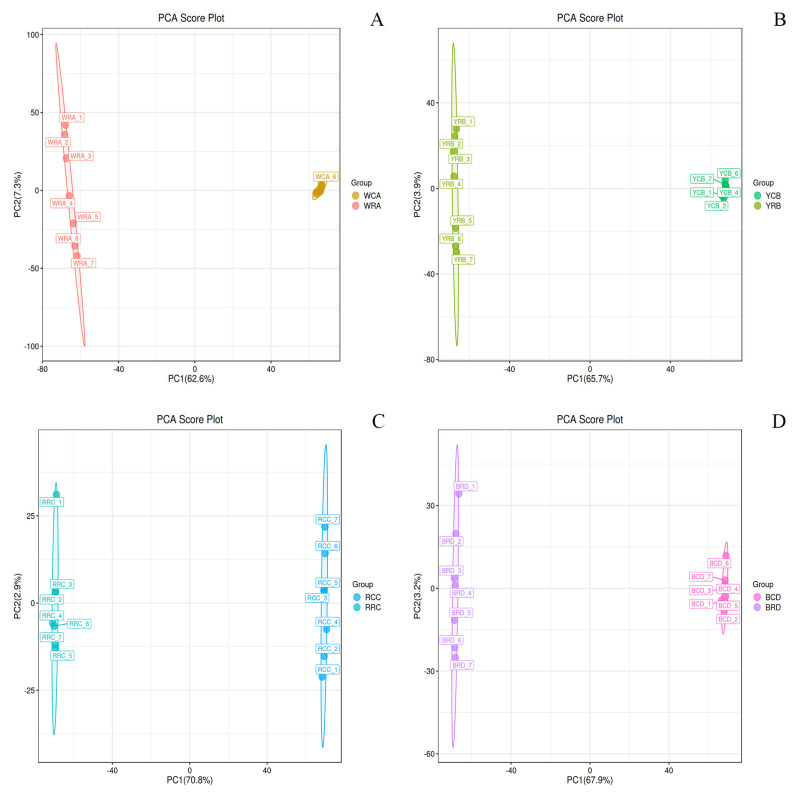
PCA Score Plots of Positive Ion Mode. (**A**–**D**) present the PCA score plots for the white, yellow, red, and black kidney bean varieties under positive ion mode, respectively (the corresponding negative ion mode PCA plots are provided as Figure S3 in the Supplementary Materials). In these figures, t[1] denotes principal component 1, and t[2] denotes principal component 2. The ellipse signifies the 95% confidence interval. Dots of the same color represent individual biological replicates within each group, and the spatial distribution of these dots indicates the extent of variation both between and within groups.

To identify biologically significant changes, we employed multivariate statistical modeling: Principal Component Analysis (PCA), an unsupervised method, first confirmed tight QC clustering (*p* < 0.05) and clear separation between treated and control groups (Figure 2 and Figure S3), validating analytical stability and treatment effects. The PLS-DA score plot revealed clear group separation, confirming distinct metabolic patterns between treatments (Figures S4 and S5).

**Figure 3:**
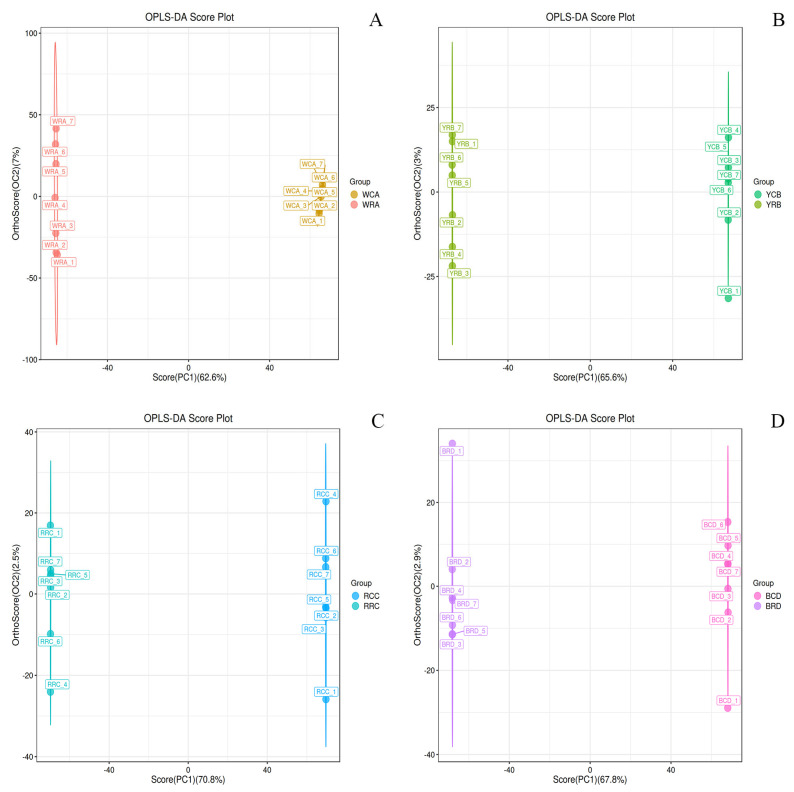
OPLS-DA Score Plots of Positive Ion Mode. (**A**–**D**) present the OPLS-DA score plots for the white, yellow, red, and black kidney bean varieties under positive ion mode, respectively (the corresponding negative ion mode OPLS-DA plots are provided as Figure S6 in the Supplementary Materials). In these figures, t[1] denotes the predictive component, and t[1] denotes the orthogonal component. The ellipse represents the 95% confidence interval. Dots of the same color correspond to individual biological replicates within the same group, and their distribution illustrates the extent of variation both between and within groups.

OPLS-DA, a supervised method, then quantified intergroup differences—all comparisons (e.g., WCA_vs_WRA) achieved perfect explained variance (R2Y = 1) and high predictive ability (Q2 ≥ 0.996; Figure 3 and Figure S6; Table 2).

**Figure 4:**
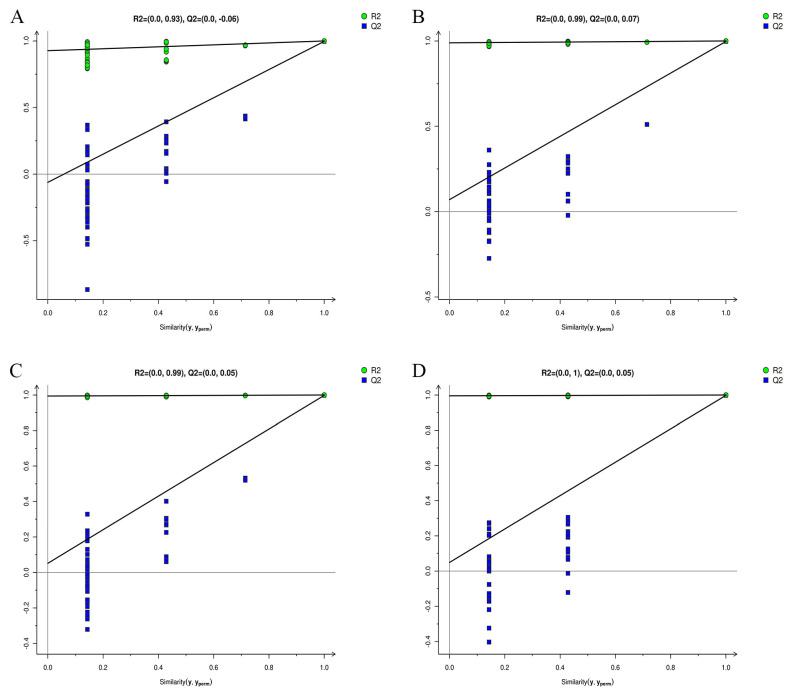
Permutation test results of the OPLS-DA models. (**A**–**D**) present the permutation test plots for the OPLS-DA models of the white, yellow, red, and black kidney bean varieties under positive ion mode, respectively (the corresponding negative ion mode permutation test plots are provided as Figure S7 in the Supplementary Materials). The permutation test was performed with 200 iterations to assess the robustness and prevent overfitting of the models. The vertical axis represents the goodness of fit (R^2^) and the predictive ability (Q^2^) of the permuted models, while the horizontal axis shows the correlation coefficient between the permuted and original class labels. The dashed lines indicate the regression lines of R^2^ (blue) and Q^2^ (green) relative to the correlation coefficient. The original R^2^ and Q^2^ values (located at the rightmost part of the plot) are significantly higher than those of the permuted models (leftward), demonstrating the validity and reliability of the original OPLS-DA models.

Model robustness was confirmed by high explained metabolic variance (R2X = 66.2–73.4%) and negative Q2 intercepts from permutation testing (Figure 4 and Figure S7), ruling out overfitting. Differentially Expressed Metabolites (DEMs) were defined using stringent criteria: VIP score > 1 (indicating strong discriminatory power), *p* < 0.05 (statistical significance), and fold change (FC) > 1.5 or <0.67 (biological relevance). This identified 291 (WCA vs. WRA), 299 (YCB vs. YRB), 320 (RCC vs. RRC), and 303 (BCD vs. BRD) differentially abundant metabolites (predominantly upregulated; Table S2). All significant metabolites were annotated using authentic standards with level 2+ confidence (MSI guidelines, Figure S8). Hierarchical clustering distinguished treatment effects (Figure S9), while biological replicates showed <30% CV for DEMs, further ensuring reliability. Phenylpropanoids (e.g., ferulic acid) were upregulated more in colored (85–92%) than white beans (78%). Primary (45%, e.g., amino acids) and secondary metabolites (35%, e.g., phenols) dominated the profile (Table S2).

### 3.3. The Effect of Microwave Treatment on the Accumulation of DEMs

**Figure 5:**
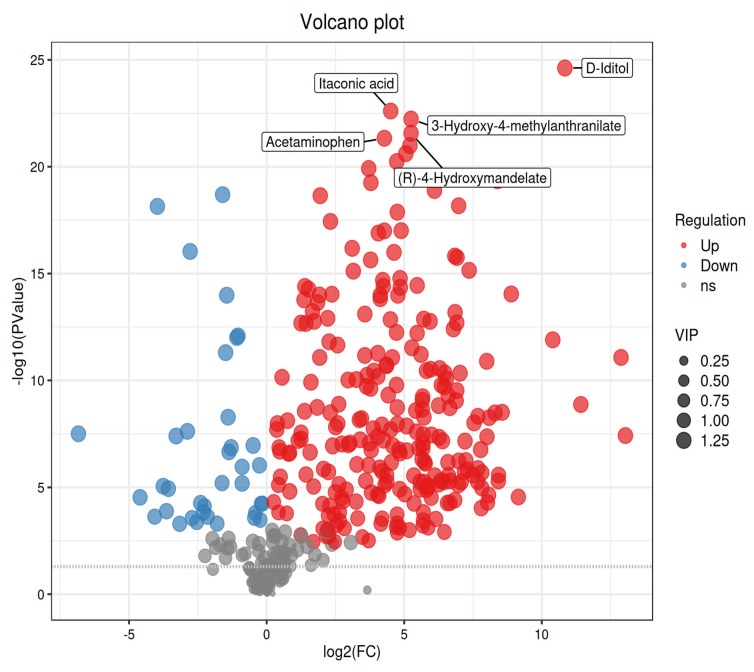
The volcano plot displays the results of differential metabolite analysis between the control and microwave-treated white kidney bean groups under positive ion mode. The *x*-axis represents the log2 fold change (log2FC) of metabolite abundance, and the *y*-axis shows the -log10 of the *p*-value from statistical testing. Significantly up-regulated metabolites (log2FC > 1, *p* < 0.05) are highlighted in red, down-regulated metabolites (log2FC < −1, *p* < 0.05) are shown in blue, and metabolites not significantly changed are indicated in gray (the corresponding volcano plots for the yellow, red, and black kidney bean varieties are provided as Figures S10 and S11, and in the Supplementary Materials, respectively).

Microwave processing induced profound metabolic alterations across kidney bean varieties as revealed by non-targeted metabolomics (Tables S1 and S2). To systematically visualize these changes, differentially expressed metabolites (DEMs) were illustrated using a volcano plot, highlighting significantly upregulated and downregulated compounds based on rigorous statistical criteria (Figure 5 and Figures S10–S12).

**Figure 6:**
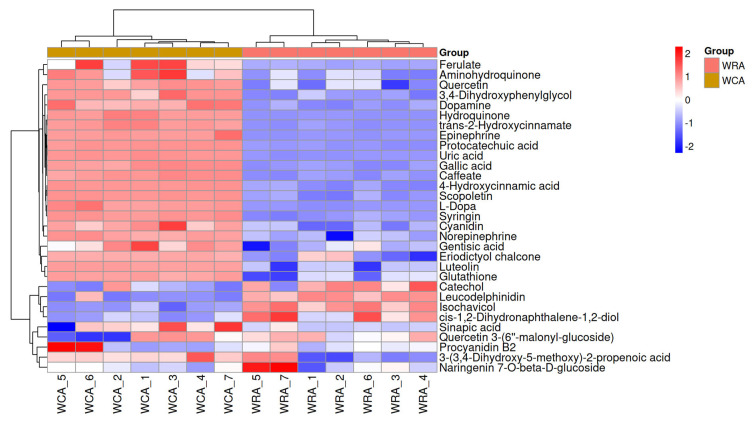
Clustering heatmap of antioxidant-related differentially accumulated metabolites in white kidney bean seeds. The heatmap displays the relative abundance patterns of antioxidant-related metabolites that were significantly altered between control and microwave-treated white kidney bean groups. Each row represents a metabolite, and each column represents a biological sample. The color scale indicates the normalized abundance levels, with red representing higher expression and blue representing lower expression. Unsupervised hierarchical clustering was applied to group metabolites and samples based on their abundance similarity (the corresponding clustering heatmaps for the yellow, red, and black kidney bean varieties are provided as Figures S13–S15 in the Supplementary Materials, respectively).

Furthermore, hierarchical clustering analysis clearly delineated treatment-specific metabolic profiles, emphasizing consistent patterns among biological replicates and distinct separation between control and microwave-treated groups (Figure 6 and Figures S13–S15). Distinct variety-specific responses were observed: White beans showed significant upregulation in monosaccharides (D-Fructose, FC = 56.12), sucrose (FC = 11.87), phenolic amines (Phenylethylamine, FC = 204.68), and flavonoids (Luteolin, FC = 51.46); yellow beans exhibited disaccharide decreases (Trehalose, FC = 0.09) contrasting with monosaccharide increases (Beta-D-Glucose, FC = 33.84), alongside exceptional upregulation of saturated fatty acids (Dodecanoic acid, FC = 620.62), phenolic amines (FC = 968.40), and glutathione (FC = 901.99); red beans displayed extreme monosaccharide elevations (Beta-D-Glucose, FC = 144.3), oligosaccharide decreases (Raffinose, FC = 0.13), lipid elevations (Dodecanoic acid, FC = 3764.22), and vitamin B6 (FC = 200.36); while black beans featured unique D-Iditol accumulation (FC = 1644.29), lipid remodeling (Dodecanoic acid, FC = 1744.53), L-Dopa (FC = 27.77), and glutathione elevation (FC = 761.77). Crucially, key commonalities underpinned detoxification: Universal hydrolytic breakdown of complex carbohydrates increased monosaccharides (D-Fructose/Beta-D-Glucose) across varieties; pronounced upregulation of saturated fatty acids (e.g., Dodecanoic acid) represented a core thermal stress response; and all varieties showed concerted induction of protective metabolites including phenolic amines (e.g., Phenylethylamine), flavonoids (Luteolin, Quercetin), phenolic acids (Gallic acid), glutathione (yellow/red/black), L-Histidine, and vitamin B6—collectively indicating activation of antioxidant systems and secondary metabolism that facilitate toxin reduction. This metabolic reprogramming mechanistically explains the observed degradation of antinutrients (Table 2) and enhanced nutritional profiles.

### 3.4. Enrichment Analysis of Metabolic Pathways of Different Metabolites

**Figure 7:**
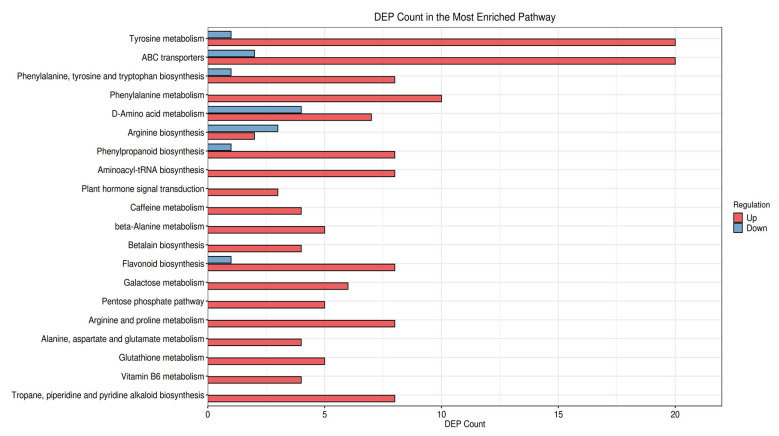
The bar plot displays the top significantly enriched KEGG pathways (*p*-value < 0.05) based on differential metabolites identified between control and microwave-treated white kidney bean groups. The *x*-axis represents the enrichment factor (rich factor) or the number of metabolites mapped to the pathway, and the *y*-axis indicates the pathway names. The length of the bars corresponds to the enrichment significance or metabolite count, and the color of the bars represents the range of *p*-values (or −log10(*p*-value)) indicating the statistical significance (the corresponding KEGG pathway enrichment analyses for the yellow, red, and black kidney bean varieties are provided as Figures S16–S18 in the Supplementary Materials, respectively).

Pathway analysis confirmed systemic metabolic reprogramming (Figure 7 and Figures S16–S18; Table S3). Universal upregulation of phenylpropanoid biosynthesis (osa00940) and phenylalanine metabolism (osa00360) across all varieties—evidenced by elevated 4-hydroxycinnamic acid (3.2–5.8-fold) and caffeic acid (2.1–4.7-fold)—directly supports enhanced tannin precursor degradation. Tyrosine metabolism (osa00350) showed pronounced changes (*p* = 2.26 × 10^−7^–7.83 × 10^−9^), while glutathione metabolism activation (osa00480; γ-glutamyl-L-cysteine +2.9–4.1-fold) suggests redox-mediated phytate degradation. ABC transporter alterations (19–26 metabolites) involved glutathione upregulation (2.3–3.6-fold).

**Figure 8:**
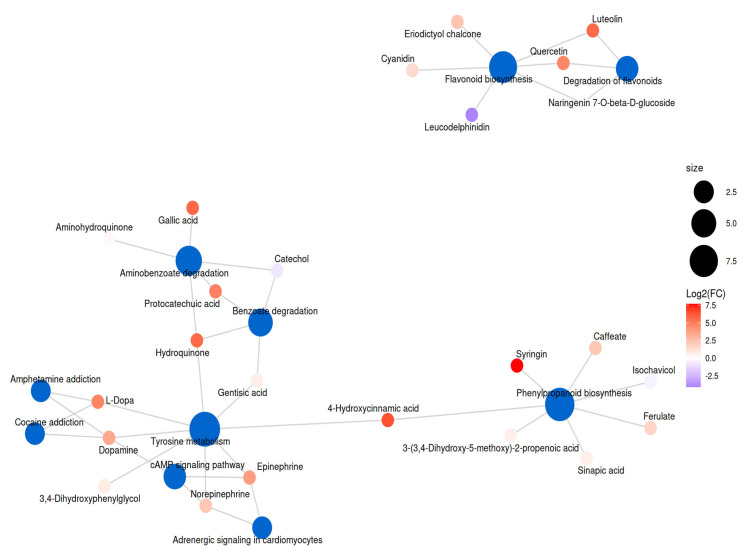
KEGG metabolic network analysis of differential metabolic pathways in white kidney bean seeds. The network illustrates the interactions among significantly enriched antioxidant-related metabolic pathways and typical antioxidant metabolites that were upregulated following microwave treatment. Nodes represent metabolic pathways, with their size and color intensity reflecting the degree of enrichment or the number of associated metabolites. Edges denote functional connections or shared metabolites between pathways. This integrative analysis highlights key metabolic processes and potential regulatory mechanisms influenced by microwave treatment in white kidney beans (corresponding KEGG metabolic network analyses for the yellow, red, and black kidney bean varieties are provided in Supplementary Materials as Figures S19–S21, respectively).

Notably, the results revealed not only a significant increase in the content of various antioxidant-related metabolites, but also the activation of their associated metabolic pathways, forming an interconnected metabolic network (Figure 8 and Figures S19–S21). Critically, saponin-associated pathways exhibited color-specific modulation: betalain biosynthesis (osa00965) upregulation in black beans (L-dopa +5.3-fold) facilitates alkaloid detoxification, while flavonoid biosynthesis (osa00941) dominance in yellow/red beans (quercetin +4.2-fold) reflects differential glycoside cleavage. This coordinated upregulation of both antioxidant metabolites and pathways underscores a robust enhancement of the antioxidant defense system following microwave treatment. Additional color-specific responses included jasmonic acid elevation (+58.6-fold) in black beans, TCA cycle downregulation in red beans (citric acid −2.1-fold), and plant hormone signaling alterations. These coordinated pathway shifts—particularly the genotype-specific induction of phenylpropanoid, glutathione, and triterpenoid metabolism—provide biochemical evidence for the microwave-induced detoxification efficacy and antioxidant accumulation reported in Table 2.

## 4. Discussion

### 4.1. Microwave Detoxification Efficacy and Mechanisms in Colored Kidney Beans

Microwave processing significantly reduced key antinutrients across all four colored kidney bean varieties (white, yellow, red, black) (Figure 1A). While microwave-only treatment achieved substantial reductions, the water soaking + microwave combination showed markedly greater efficacy, successfully reducing all antinutrients below established safety thresholds (phytic acid < 5 mg/g DW, tannins < 1 mg/g DW, saponins < 3 mg/g DW) (Figure 1B–D), aligning with clinical safety limits for legume consumption [4,14]. Black beans consistently exhibited higher residual antinutrient levels despite treatment, a phenomenon attributable to two counteracting factors: (1) their dense phenolic matrix and thicker seed coat structure (38% thicker than white beans per [32]) inherently impede water penetration, resulting in their lowest water absorption capacity (9.3% ± 0.6%) during the 5 min prehydration, and (2) the limited hydration consequently restricted hydration-mediated thermal transfer and leaching of water-soluble antinutrients [32,33]. This observation is further corroborated by Manikpuri et al. [34], who reported that dense, lignin-rich matrices significantly impede phytate and tannin degradation during microwave processing.

Our results align with previous studies on microwave processing [35] but reveal critical distinctions that underscore the novelty of our combined approach. The key advancement is demonstrating that a brief hydration pre-treatment dramatically potentiates microwave efficacy, reducing processing time by 80% compared to conventional boiling [36] while achieving safety thresholds. This synergistic effect contrasts with dry heat methods [6] and emphasizes the critical role of water, as recently shown in sesame seeds [10]. Furthermore, while microwave pretreatment enhances oil extraction in oilseeds [9], our work establishes a rapid, water-assisted protocol specifically optimized for legume detoxification.

The water absorption hierarchy directly modulated detoxification efficacy through three synergistic mechanisms: (1) enhanced microwave heat penetration through pre-hydrated tissues [9], (2) partial leaching of water-soluble antinutrients during soaking [10], and (3) microwave-induced structural disruption of hydrated cellular matrices [34]. Water absorption likely weakened hydrogen bonds in phytic acid-mineral complexes and increased tannin solubility [7], with varieties showing higher hydration demonstrating superior detoxification. The dramatic tannin reduction reflects their heat-labile nature and water solubility [5]. The differential reduction across bean colors stems from fundamental biochemical and structural disparities inherent to their seed coats [1,15,33]. The superior detoxification in white beans aligns with their highest water absorption and less complex structure [5,10], while higher residual levels in black beans result from their dense phenolic matrix [1,33] that likely forms stable complexes with antinutrients [15,34].

Metabolomic evidence suggests that microwave treatment activates dual detoxification pathways: direct thermal degradation and induction of endogenous detoxification systems. We observed upregulation of glutathione metabolism (γ-glutamyl-L-cysteine +2.1–3.8-fold) and phenylpropanoid biosynthesis (4-hydroxycinnamic acid +2.9–4.5-fold), indicating activated cellular defense mechanisms that potentially facilitate sequestration of toxic metal ions [37] and may reinforce cell walls (Tables S2 and S3). The mechanism involves microwave dielectric heating disrupting molecular bonds [7,34], while water soaking may preserve metabolic enzymes through thermal buffering [9,32].

Collectively, our findings establish an ultra-rapid, water-assisted microwave protocol that achieves complete detoxification in merely 5 min, representing a paradigm shift from conventional methods. The mechanistic revelation that this synergy is directly modulated by the seed’s water absorption hierarchy, influenced by seed coat morphology, provides a new predictive model for optimizing processing conditions across diverse legume varieties.

### 4.2. Microwave-Enhanced Antioxidant Accumulation and Underlying Mechanisms

Microwave processing significantly enhanced antioxidant capacity across all kidney bean varieties, with water soaking pretreatment demonstrating markedly superior efficacy over microwave treatment alone (Figure 1E, Figure 6 and Figures S13–S15; Table 3). The observed color-dependent hierarchy (black/red > yellow > white) confirms intrinsic linkages between seed pigmentation and antioxidant potential [1,38], aligning with findings that dark-seeded genotypes inherently possess higher phenolic content [1]. The dramatic FRAP elevation in red beans correlates with their anthocyanin-rich composition [33], consistent with reports that pigmented cultivars exhibit superior antioxidant responsiveness under thermal stress.

Our results extend significantly beyond existing literature [35,37], as the profound boost achieved by our combined method demonstrates clear advantages over previous techniques. This aligns with recent work showing hydration–microwave synergies in other seeds [10,39], though our protocol achieves rapid effects without germination. Crucially, our metabolomic data provides mechanistic depth often missing in prior studies (Figure 6, Figure 7 and Figures S13–S15). The upregulation of phenylpropanoid biosynthesis mirrors observations in microwave-processed buckwheat [40] but we directly link this activation to pre-hydration’s protective effect. The exceptional preservation of glutathione in pre-soaked beans represents a novel finding, as the specific role of hydration in preserving heat-labile antioxidants via thermal buffering has not been previously detailed in legume microwave processing [39]. Our study thus not only confirms microwave enhancement of antioxidant properties but mechanistically demonstrates how pre-soaking maximizes this benefit, refining understanding of microwave applications in food biofortification. The color-dependent antioxidant response is further supported by recent investigations establishing clear correlations between seed coat pigmentation and intrinsic antioxidant capacity [38].

Metabolomic analysis revealed three key mechanisms driving antioxidant enhancement (Table 3, Tables S2 and S3; Figure 8 and Figures S19–S21): First, microwave-induced cell wall disruption [41] liberated bound phenolics through rapid vaporization of intracellular water, creating internal pressure that ruptures cellular structures [9]. Second, water soaking preserved heat-labile antioxidants by reducing thermal degradation [32], with water’s high specific heat capacity buffering against temperature spikes and protecting enzymatic machinery. Third, microwaves redirected metabolic flux toward phenylpropanoid biosynthesis [10], a phenomenon amplified by water pretreatment’s hydration of enzymatic substrates.

Ferric reducing antioxidant capacity and DPPH radical scavenging data corroborated these findings (Figure 1E,F), showing the combined treatment nearly doubled the improvement compared to microwave alone. The strong response in red beans reflects their high flavonoid content, while white beans exhibited compensatory antioxidant induction through tyrosine metabolism [42].

Collectively, our evidence reveals that water soaking potentiates microwave effects through three interconnected mechanisms: enhanced dielectric heating efficiency, protection of enzymatic systems [32,42], and facilitated antioxidant mobilization. These synergistic processes explain the substantially greater antioxidant enhancements versus microwave-only treatment, while the protocol’s efficiency and cross-varietal efficacy confirm its industrial viability.

The most significant insight is the mechanistic demonstration that brief water soaking serves a dual protective and enhancing role: it mitigates thermal degradation of heat-labile antioxidants while potentiating microwave-induced upregulation of biosynthetic pathways (Figure 8 and Figures S19–S21). This finding moves beyond established knowledge by elucidating how pre-hydration protects and amplifies metabolic pathways, positioning the soaking-microwave combination as an efficient strategy for targeted legume biofortification.

### 4.3. Microwave Induced Other Bioactive Metabolite Enrichment and Mechanisms

Microwave processing induced significant, color-dependent accumulation of non-antioxidant bioactive metabolites across all varieties (Table 3, Tables S2 and S3). Gamma-aminobutyric acid (GABA), beneficial for neurological health [43], increased most in black (FC = 6.59, FDR = 4.10 × 10^−21^) and white beans (FC = 4.51, FDR = 0.003), consistent with the report by Hou et al. [43] that microwave irradiation enhances glutamate decarboxylase activity—the key enzyme in GABA shunt pathway. L-Carnitine (fatty acid metabolism; Sawicka et al., [44]) surged in red beans (FC = 65.02, FDR = 1.15 × 10^−12^), reflecting preferential activation of mitochondrial β-oxidation pathways in pigmented varieties, while dopamine (immune modulation; Franco et al. [45]) peaked in red beans (FC = 130.05, FDR = 6.55 × 10^−13^), indicating tyrosine hydroxylase upregulation under microwave stress. Spermidine (longevity; Zou et al. [46]) increased universally (white FC = 2.86; black FC = 2.09), aligning with the observation by Zou et al. [46] that thermal processing enhances arginine decarboxylase-mediated polyamine biosynthesis. β-vitamins showed color-specific responses: pyridoxal (B6; [47]) surged in yellow beans (FC = 37.61, FDR = 4.48 × 10^−13^), while pantothenic acid (B5; [48]) increased most in white beans (FC = 13.71, FDR = 7.79 × 10^−15^), demonstrating genotype-specific vitamin stability during microwave exposure. Cyclic AMP (immune regulation; [49]) was highly upregulated in white (FC = 59.45, FDR = 2.31 × 10^−5^) and yellow beans (FC = 61.02, FDR = 3.11 × 10^−14^), suggesting adenylate cyclase activation in low-pigment varieties as a compensatory signaling mechanism.

Pathway analysis revealed fundamental divergences (VIP > 0.7, FDR < 0.05): While amino acid metabolism was universally enhanced (driving GABA, spermidine, dopamine), red/black beans prioritized lipid metabolism (modulating L-carnitine), corroborating the findings of Ghallab et al. [50] on pigment-linked metabolic specialization in kidney beans. White/yellow beans favored vitamin cofactor metabolism (pyridoxamine: white FC = 2.62; yellow FC = 3.11) and signal transduction (acetylcholine/cAMP), consistent with the observation by Bento et al. [51] that light-colored legumes amplify vitamin retention during processing. This selective pathway modulation demonstrates microwave’s ability to tailor health-promoting metabolite profiles based on bean color, as proposed in the seed coat color-metabolome correlation model by Li et al. [52]. Furthermore, a very recent, comprehensive metabolomics study by Ghallab et al. [50] on processed red kidney beans also revealed that processing methods significantly alter the anti-diabetic metabolite profiles in a way that is influenced by the bean’s inherent chemical composition, which is often linked to seed coat color, thus providing independent validation for our core finding of color-dependent metabolic rewiring. Similarly, Ahmed et al. [53] demonstrated that both water bath and ultrasonic pretreatments significantly increased the content of key bioactive compounds such as tocopherols and phenolic substances in pumpkin seeds, with further enhancement observed after roasting. This supports our observation that appropriate physical processing can promote the accumulation of health-beneficial metabolites. Notably, the study attributed the increase to mechanisms such as the release of bound phenolics and the improvement of extractability due to cellular structure disruption, which parallels our proposed mechanism of microwave-induced “carbon sink” effect that redirects metabolic flux toward bioactive synthesis. The metabolic rewiring—suppression of primary metabolism (TCA cycle intermediates such as citrate and succinate decreased 1.5–2.0 fold) and upregulation of specialized pathways—creates a “carbon sink” effect that redirects resources toward targeted bioactive synthesis, facilitating enrichment of non-antioxidant bioactives and expanding microwave’s nutritional benefits beyond detoxification and antioxidant enhancement (Figure 7, Figure 8 and Figures S16–S21). The pronounced accumulation of GABA and dopamine in this stress-response metabolome aligns with a proposed shutdown of primary energy metabolism to conserve ATP, channeling amino acid precursors like glutamate and tyrosine into secondary, stress-induced neuroactive compounds [43,45].

### 4.4. Limitations and Future Perspectives

This study successfully demonstrates that microwave processing effectively reduces antinutrients while enhancing antioxidant capacity in colored kidney beans, with water soaking pretreatment proving particularly advantageous by improving detoxification efficiency by 2.1–2.8-fold and antioxidant levels by 65.9–115.2% compared to dry microwave treatment. The observed inverse relationship between antinutrient reduction and antioxidant accumulation is intrinsically linked to the metabolic pathway regulation induced by the combined treatment, wherein the degradation of antinutritional complexes and the liberation and biosynthesis of antioxidants are facilitated concurrently. A holistic visual representation of these interconnected mechanisms would indeed provide a more comprehensive interpretation. Accordingly, we will incorporate a new schematic diagram as a Supplementary Table (Table S4) to illustrate this synergistic process. However, several limitations reveal critical research gaps that simultaneously define future investigation priorities. From an industrial scalability perspective, the proposed water-assisted microwave protocol shows significant promise. The 80% reduction in processing time compared to conventional boiling [36] translates directly to higher throughput and lower energy costs per unit mass in a continuous flow system, as recently demonstrated in the development of energy-efficient drying methods for legumes [12]. The minimal equipment requirement—essentially a soaking vessel and an industrial microwave tunnel—suggests a relatively low capital investment barrier. However, economic viability hinges on optimizing energy and water consumption. The trade-off between reduced thermal energy input and increased water usage presents a key sustainability consideration, necessitating life cycle assessment (LCA) studies to quantify the net environmental impact and operational costs. Furthermore, the potential for water recycling within the process must be evaluated to minimize waste, potentially drawing on innovations in ultrasonic-assisted soaking processes [11]. The impact on product quality is another critical factor for adoption. Our results demonstrate superior retention and enhancement of antioxidants compared to traditional methods, which is a significant market advantage for health-conscious consumers. However, industrial application requires consistent quality across large batches. The observed color-dependent efficacy, which is consistent with findings in other legumes such as soybeans [2] and azuki beans [38], necessitates the development of variety-specific processing parameters to ensure uniform detoxification and nutrient enhancement, which could be achieved through automated sorting systems and adaptive process control. Finally, comprehensive sensory evaluation and shelf-life studies under commercial packaging conditions are essential next steps to confirm that the improved nutritional profile translates into consumer acceptance and stable quality during distribution. The most pressing uncertainty involves water’s synergistic mechanisms—while we observed significantly improved outcomes with hydration pretreatment, the exact relationships between soaking parameters (time/temperature/water quality) and dielectric properties remain unclear, necessitating systematic studies using advanced imaging techniques to map water distribution patterns during processing, as recently pioneered in sesame seeds [10] and other agricultural products. The striking color-dependent responses, where red varieties outperformed white by 45% in antioxidant enhancement, point to another key knowledge gap regarding how seed coat microstructure and pigment composition influence microwave sensitivity—a question ideally addressed through comparative micro-CT analyses of cellular architecture as demonstrated in lotus seeds [32], avoiding enzymatic approaches while still elucidating structural determinants of varietal differences. This is further supported by recent metabolomic studies highlighting the profound impact of processing on the phytochemical profile of colored beans [50,51]. Technical limitations in current protocols simultaneously highlight three translational research needs: (1) industrial optimization requiring determination of minimum hydration times and energy inputs for commercial-scale equipment, building on sesame oil processing models [10] and recent legume processing studies [34]; (2) comprehensive evaluation of nutrient retention during storage, particularly for anthocyanins in pigmented varieties, as color stability is a known challenge [52]; and (3) sensory impact assessments of water-assisted processing on texture and flavor profiles. The restricted scope of distilled water use in this study further suggests that investigating modified soaking solutions (mineral-enriched/acidic) could yield additional benefits, as evidenced by recent work in sesame seeds [10], while the fixed 5 min microwave duration calls for response surface methodology to establish variety-specific optima. Broader applicability questions demand multispecies validation across legumes like azuki beans [38] and soybeans [42] to test whether seed coat pigmentation universally predicts microwave responsiveness. Recent studies on microwave pretreatment in various seeds [8,40] suggest that the activation of biosynthetic pathways may be a general phenomenon, warranting further investigation. Practical implementation challenges, particularly the trade-off between the protocol’s 80% time savings over conventional boiling [36] versus increased water consumption, present sustainability considerations requiring innovations like water recycling systems. Collectively, these limitations not only constrain current interpretation but strategically map the essential next steps for developing microwave processing into a robust, scalable technology for legume quality improvement.

## 5. Conclusions

This study demonstrates that intermittent microwave processing, particularly when preceded by a brief water soaking pretreatment, effectively reduces key antinutrients (phytic acid, tannins, and saponins) below safety thresholds while significantly enhancing antioxidant capacity across four colored kidney bean varieties. The combined water soaking and microwave treatment yielded the most substantial antinutrient reduction and highest increase in FRAP values, with red kidney beans showing the strongest response. Metabolomic analysis revealed that this combined approach upregulates phenylpropanoid and glutathione biosynthesis pathways, promoting bioactive compound accumulation.

However, the precise role of water in enhancing dielectric properties and the structural basis for color-dependent effects require further investigation. Future studies should employ micro-CT imaging and optimized hydration protocols to elucidate water distribution and cellular disruption mechanisms. Additionally, industrial-scale validation, cost–benefit analysis, and sensory evaluations are necessary to assess practical applicability.

The proposed method reduces processing time by approximately 80% compared to conventional boiling, offering a scalable and energy-efficient alternative for legume preprocessing. Theoretically, this work highlights microwave-induced metabolic shifts that concurrently improve safety and nutritional quality, with seed color potentially predicting efficacy. Further research should validate the method’s transferability to other legumes and assess nutrient stability and bioavailability in vivo.

In summary, this study provides an efficient, mechanistically grounded processing strategy for developing safer and more nutritious legume-based foods with significant industrial potential.

## Figures and Tables

**Table 1:** Metabolite analysis in positive and negative ion modes.

	Total Metabolites	Up	Down	Total DE
WCA/WRA (P)	7374	3792	621	4413
WCA/WRA (N)	8842	3870	1176	5046
YCB/YRB (P)	7374	3555	986	4541
YCB/YRB (N)	8842	3443	1622	5065
RCC/RRC (P)	7374	3685	1201	4886
RCC/RRC (N)	8842	3667	1775	5442
BCD/BRD (P)	7374	3754	931	4685
BCD/BRD (N)	8842	3812	1232	5044

Note: P, positive ion mode; N, negative ion mode.

**Table 2:** Evaluation of OPLS-DA model quality for discrimination between control and microwave-treated kidney bean groups across different varieties and ion modes. The table summarizes key model parameters including the number of predictive and orthogonal components (pre), the cumulative explained variance of the X-matrix (R2X(cum)), the cumulative modeled variation in the response Y (R2Y(cum)), and the cumulative predictive ability (Q2(cum)) assessed by cross-validation. Sample group abbreviations represent comparisons between control and microwave-treated samples for each variety. High R2Y and Q2 values to 1 indicate excellent explanatory power and predictive robustness of all models, with no signs of overfitting.

Ionic Mode	Sample Groups	pre	R^2^X (cum)	R^2^Y (cum)	Q^2^ (cum)
Positive ion mode (POS)	WCA_vs_WRA	1 + 1 + o	0.696	1	0.997
Negative ion mode (NEG)	WCA_vs_WRA	1 + 1 + o	0.672	1	0.997
Positive ion mode (POS)	YCB_vs_YRB	1 + 1 + o	0.687	1	0.997
Negative ion mode (NEG)	YCB_vs_YRB	1 + 1 + o	0.664	1	0.997
Positive ion mode (POS)	RCC_vs_RRC	1 + 1 + o	0.734	1	0.998
Negative ion mode (NEG)	RCC_vs_RRC	1 + 1 + o	0.72	1	0.998
Positive ion mode (POS)	BCD_vs_BRD	1 + 1 + o	0.708	1	0.998
Negative ion mode (NEG)	BCD_vs_BRD	1 + 1 + o	0.662	1	0.996

**Table 3:** Microwave significantly up-regulates antioxidant differential metabolites (FDR < 0.05, VIP > 1).

Num	DEMs	Fold Change (FC) Values			
		WCA/WRA	YCB/YRB	RCC/RRC	BCD/BRD
1	Quercetin	26.64	13.75	83.72	30.89
2	Cyanidin	2.69	38.82	106.38	5.4
3	Luteolin	51.46	142.74	94.42	106.92
4	Gallic acid	52.33	101.74	132.34	78.04
5	Caffeate (Caffeic acid)	4.65	5.71	4.63	5.13
6	Protocatechuic acid	29.64	19.67	37.66	18.03
7	Glutathione (GSH)	51.13	901.99	171.47	761.77
8	Dopamine	10.92	36.28	130.05	8.92
9	Epinephrine	14.72	22.96	89.83	23.1
10	Norepinephrine	4.73	5.66	26.63	13.66
11	Hydroquinone	52.82	72.38	156.37	44.56
12	Ferulate (Ferulic acid)	3.24	2.28	11.66	2.76
13	Sinapic acid	-	-	4.79	2.33
14	3,4-Dihydroxyphenylglycol	1.7	-	4.89	4.11
15	trans-2-Hydroxycinnamate	11.89	10.34	25.41	9.62
16	4-Hydroxycinnamic acid	83.88	73.35	193.53	43.15
17	Uric acid	19.44	14.93	46.51	26.41
18	L-Dopa	27	31.21	93.14	27.77
19	Eriodictyol chalcone	-	0.64	4.53	5.04
20	Scopoletin	13.9	5.28	26.79	6.86
21	Syringin	205.23	12.72	17.73	489.09

## Data Availability

The original contributions presented in the study are included in the article/Supplementary Material. Further inquiries can be directed to the corresponding authors.

## Supplementary Materials

The following supporting information can be downloaded at: https://www.mdpi.com/article/10.3390/foods14203557/s1, Table S1. Global metabolite profile; Table S2. Secondary differential metabolites; Table S3. KEGG metabolic pathways; Table S4. Comparative Analysis of Antinutrient Reduction and Antioxidant Enhancement; Figure S1. Base peak chromatogram and total ion chromatogram. Representative LC-MS chromatograms of kidney bean extracts: (A) Base peak chromatogram (BPC, positive mode), (B) BPC (negative mode), (C) Total ion chromatogram (TIC, positive mode), and (D) TIC (negative mode); Figure S2. PCA analysis of QC and QA samples. PCA score plots of quality control samples in non-targeted metabolomics analysis of four-colored kidney beans. (A) QA samples (positive mode), (B) QA samples (negative mode), (C) QC samples (positive mode), (D) QC samples (negative mode); Figure S3. PCA Score Plots of Positive Ion Mode. (A–D) present the PCA score plots for the white, yellow, red, and black kidney bean varieties under negative ion mode, respectively. In these figures, t[1] denotes principal component 1, and t[2] denotes prin-cipal component 2. The ellipse signifies the 95% confidence interval. Dots of the same color represent individual biological replicates within each group, and the spatial distribution of these dots indicates the extent of variation both between and within groups; Figure S4. PLS-DA analysis. PLS-DA score plots of raw and microwaved green beans in positive (A–D) ion modes: white (A), yellow (B), red (C), and black (D) seed varieties; Figure S5. PLS-DA analysis. PLS-DA score plots of raw and microwaved green beans in negative (A–D) ion modes: white (A), yellow (B), red (C), and black (D) seed varieties; Figure S6. OPLS-DA Score Plots of Nega-tive Ion Mode. (A–D) present the OPLS-DA score plots for the white, yellow, red, and black kidney bean varieties under negative ion mode, respectively. In these figures, t[1] denotes the predictive component, and to[1] denotes the orthogonal component. The ellipse represents the 95% con-fidence interval. Dots of the same color correspond to individual biological replicates within the same group, and their distribution illustrates the extent of variation both between and within groups; Figure S7. Permutation test results of the OPLS-DA models. (A–D) present the permutation test plots for the OPLS-DA models of the white, yellow, red, and black kidney bean varieties under negative ion mode, respectively. The permutation test was performed with 200 iterations to as-sess the robustness and prevent overfitting of the models. The vertical axis represents the goodness of fit (R^2^) and the predictive ability (Q^2^) of the permuted models, while the horizontal axis shows the correlation coefficient between the permuted and original class labels. The dashed lines indicate the regression lines of R^2^ (blue) and Q^2^ (green) relative to the correlation coefficient. The original R^2^ and Q^2^ values (located at the rightmost part of the plot) are signif-icantly higher than those of the permuted models (leftward), demonstrating the validity and reliability of the original OPLS-DA models; Figure S8. Identification of key differential metabo-lites using authentic standards. Chromatographic match confirms annotation of (A) Jasmonic acid; (B) Adenosine; (C) Glutathione; (D) L-Arginine; (E) L-Glutamic acid; (F) Spermidine; Figure S9. Clustering heatmap. Hierarchical clustering heatmaps of metabolic profiles in raw and microwaved kidney beans: white (A), yellow (B), red (C), and black (D) seed varieties; Figure S10–S12. The volcano plot displays the results of differential metabolite analysis between the control and microwave-treated yellow (Figure S10), red (Figure S11) and black(Figure S12) kidney bean groups under positive ion mode. The x-axis represents the log2 fold change (log2FC) of metabolite abundance, and the y-axis shows the -log10 of the *p*-value from statistical testing. Significantly up-regulated metabolites (log2FC > 1, *p* < 0.05) are highlighted in red, down-regulated metabolites (log2FC < −1, *p* < 0.05) are shown in blue, and metabolites not sig-nificantly changed are indicated in gray. (The corresponding volcano plots for the yellow, red, and black kidney bean varieties are provided as Figures S10 and S11, and in the Supplementary Materials, respectively); Figure S13–S15. Clustering heatmap of antioxidant-related differentially accumulated metabolites in yellow, red and black kidney bean seeds. The heatmap displays the relative abundance patterns of antioxidant-related metabolites that were significantly altered between control and microwave-treated yellow (Figure S13), red (Figure S14) and black (Figure S15) kidney bean groups. Each row represents a metabolite, and each column represents a bi-ological sample. The color scale indicates the normalized abundance levels, with red repre-senting higher expression and blue representing lower expression. Unsupervised hierarchical clustering was applied to group metabolites and samples based on their abundance similarity; Figure S16–S18. The bar plot displays the top significantly enriched KEGG pathways (*p*-value < 0.05) based on differential metabolites identified between control and microwave-treated yellow (Figure S19), red (Figure S20) and black (Figure S21) kidney bean groups. The x-axis represents the enrichment factor (rich factor) or the number of metabolites mapped to the pathway, and the y-axis indicates the pathway names. The length of the bars corresponds to the enrichment significance or metabolite count, and the color of the bars represents the range of *p*-values (or -log10 (*p*-value)) indicating the statistical significance; Figure S19–S21. KEGG metabolic network analysis of differential metabolic pathways in kidney bean seeds. The network illustrates the in-teractions among significantly enriched antioxidant-related metabolic pathways and typical antioxidant metabolites that were upregulated following microwave treatment. Nodes repre-sent metabolic pathways, with their size and color intensity reflecting the degree of enrichment or the number of associated metabolites. Edges denote functional connections or shared me-tabolites between pathways. This integrative analysis highlights key metabolic processes and potential regulatory mechanisms influenced by microwave treatment in yellow (Figure S19), red (Figure S20) and black (Figure S21) kidney beans.

## Abbreviations

DEMs	Differentially Expressed Metabolites
DPPH	2,2-Diphenyl-1-picrylhydrazyl
DW	Dry Weight
FC	Fold Change
FDR	False Discovery Rate
FRAP	Ferric Reducing Antioxidant Power
GABA	Gamma-Aminobutyric Acid
LC-MS	Liquid Chromatography–Mass Spectrometry
MS	Mass Spectrometry
OPLS-DA	Orthogonal Partial Least Squares–Discriminant Analysis
PCA	Principal Component Analysis
WRA	White Raw Bean (Group A)
WCA	White Combined-process Bean (Group A)
YRB	Yellow Raw Bean (Group B)
YCB	Yellow Combined-process Bean (Group B)
RRC	Red Raw Bean (Group C)
RCC	Red Combined-process Bean (Group C)
BRD	Black Raw Bean (Group D)
BCD	Black Combined-process Bean (Group D)
DPPH	diphenylpicrylhydrazine
VIP	Variable Importance in Projection
