# Supplementary material for: Water-Assisted Microwave Processing: Rapid Detoxification and Antioxidant Enhancement in Colored Kidney Beans
Source: Foods. 2025 Oct 18;14(20):3557. doi: 10.3390/foods14203557 (PMC12564649; doi:10.3390/foods14203557)
Supplement: Supplementary file 1 [file foods-14-03557-s001.zip › Table S4.pdf]

Table S4 The combined treatment lowers antinutrients and raises antioxidants

Water Soaking + Microwave vs. Water Control	
**Mechanism for anti-nutrient reduction**	**Mechanism for antioxidant enhancement**
**1. Physical Leaching:** Water-soluble antinutrients (e.g., tannins) are partially dissolved and leach out into the water during the soaking stage.	**1. Liberation of Bound Phenolics:** Microwave-induced rupture of cell walls (e.g. pectin depolymerization) and subcellular structures releases bound phenolic antioxidants from cellular matrices.
2. **Enhanced Thermal Degradation & Structural Disruption:** Water penetration pre-hydrates tissues, drastically improving microwave dielectric heating efficiency. Rapid oscillation of absorbed H_2O molecules generates intense internal friction/heat. This thermal energy disrupts H-bonds in phytic acid-mineral complexes & protein-tannin conjugates, while simultaneously rupturing cell walls & subcellular structures (e.g., protein bodies, vacuoles). This dual action (bond breaking + physical rupture) degrades & releases bound antinutrients (e.g., tannins bound to proteins, phytic acid).	**2. Protection of Heat-Labile Antioxidants:** Water pretreatment acts as a "thermal buffer." Water's high specific heat capacity buffers against instantaneous temperature spikes, protecting the activity of key enzymes like glutathione reductase, thereby preserving heat-labile antioxidants such as glutathione.
**3. Chemical Hydrolysis:** Thermal energy from microwaves catalyzes the hydrolysis of ester bonds in phytic acid and glycosidic linkages in saponins, leading to their degradation.	**3. Upregulation of Antioxidant Synthesis Pathways:** Microwave stress signals upregulate phenylpropanoid biosynthesis (4-hydroxycinnamic acid +2.9–4.5 fold), channeling carbon skeletons from primary metabolism into the synthesis of phenolic acids, flavonoids, and other antioxidants. Hydration amplifies this effect by providing sufficient enzymatic substrates.
**4. Activation of Endogenous Detoxification Systems:** Microwave acts as a stressor, activating the seed's innate detoxification metabolic pathways, such as glutathione metabolism (synthesizing more glutathione to chelate toxic metal ions liberated from phytic acid) and phenylpropanoid biosynthesis (whose upstream products can also be used to synthesize lignin, reinforcing cell walls to prevent nutrient leaching).	**4. Compensatory Induction:** Even in white beans lacking pigmentation precursors, antioxidant synthesis is induced via alternative pathways like tyrosine metabolism (L-dopa +6.8 fold) in response to stress.
