# Supplementary figures and images for: Water-Assisted Microwave Processing: Rapid Detoxification and Antioxidant Enhancement in Colored Kidney Beans
Source: Foods. 2025 Oct 18;14(20):3557. doi: 10.3390/foods14203557 (PMC12564649; doi:10.3390/foods14203557)

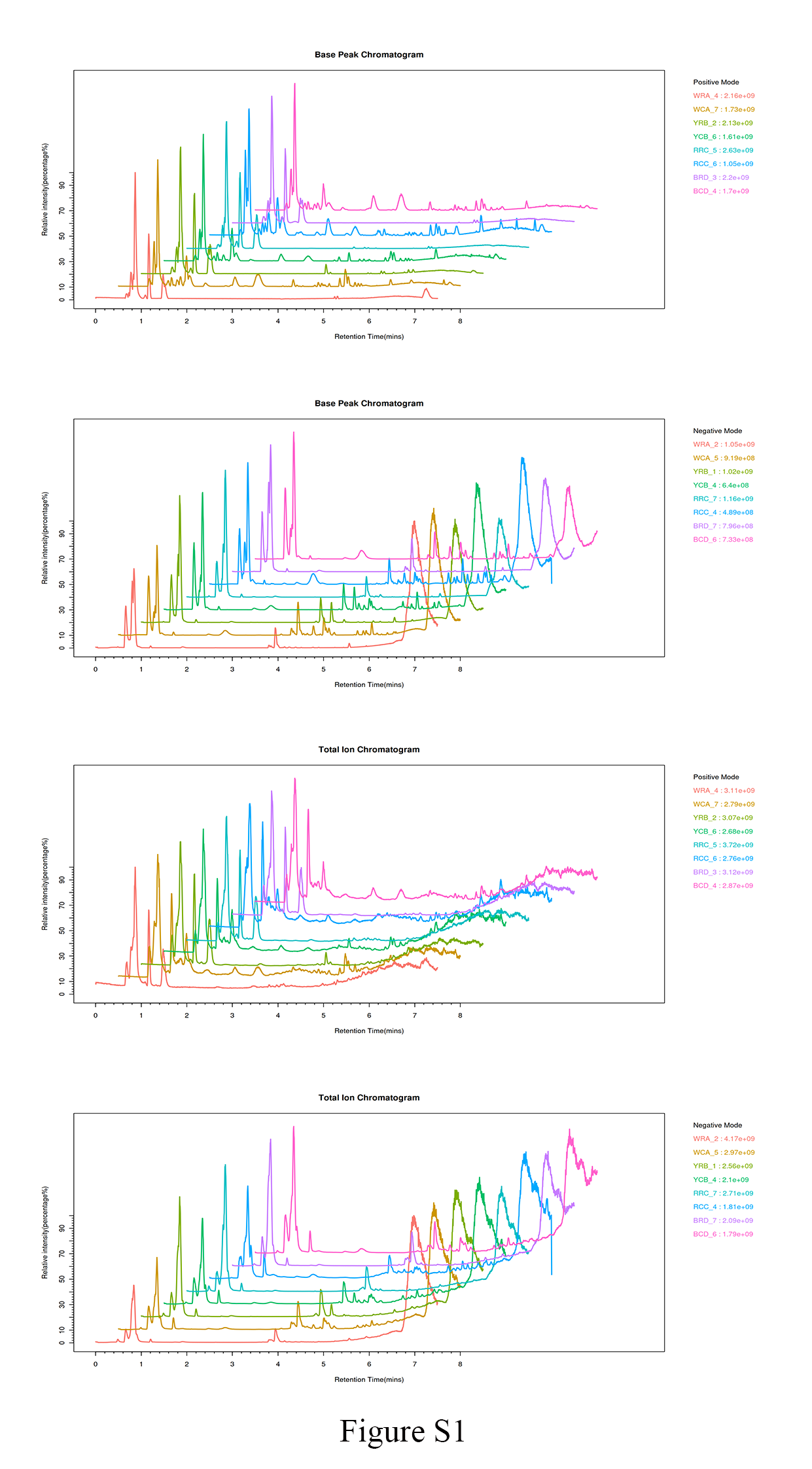

Supplement: Supplementary file 1 [file foods-14-03557-s001.zip › Figure S1 TIC-BPC.tif]

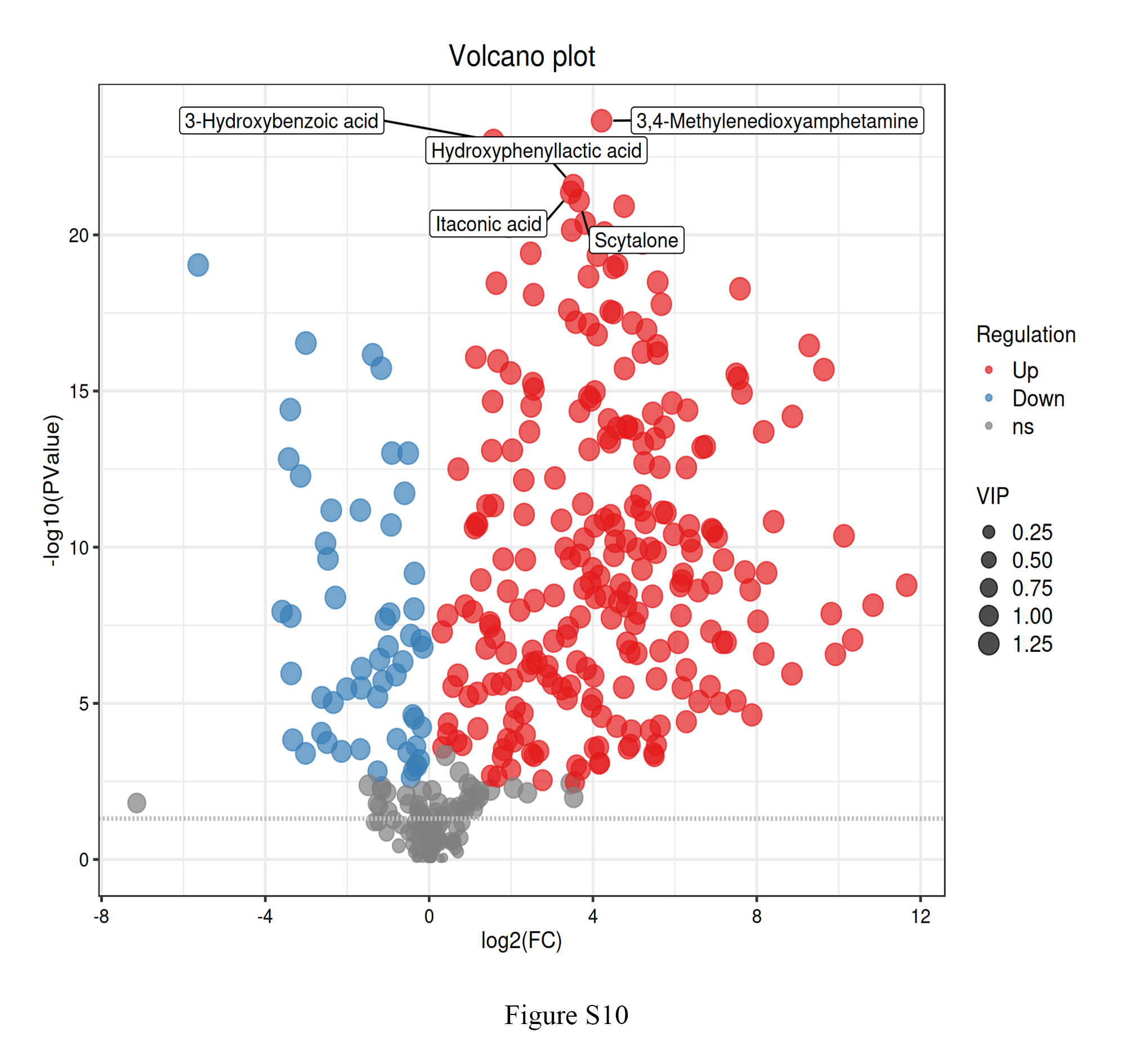

Supplement: Supplementary file 1 [file foods-14-03557-s001.zip › Figure S10 volcano yellowseed.tif]

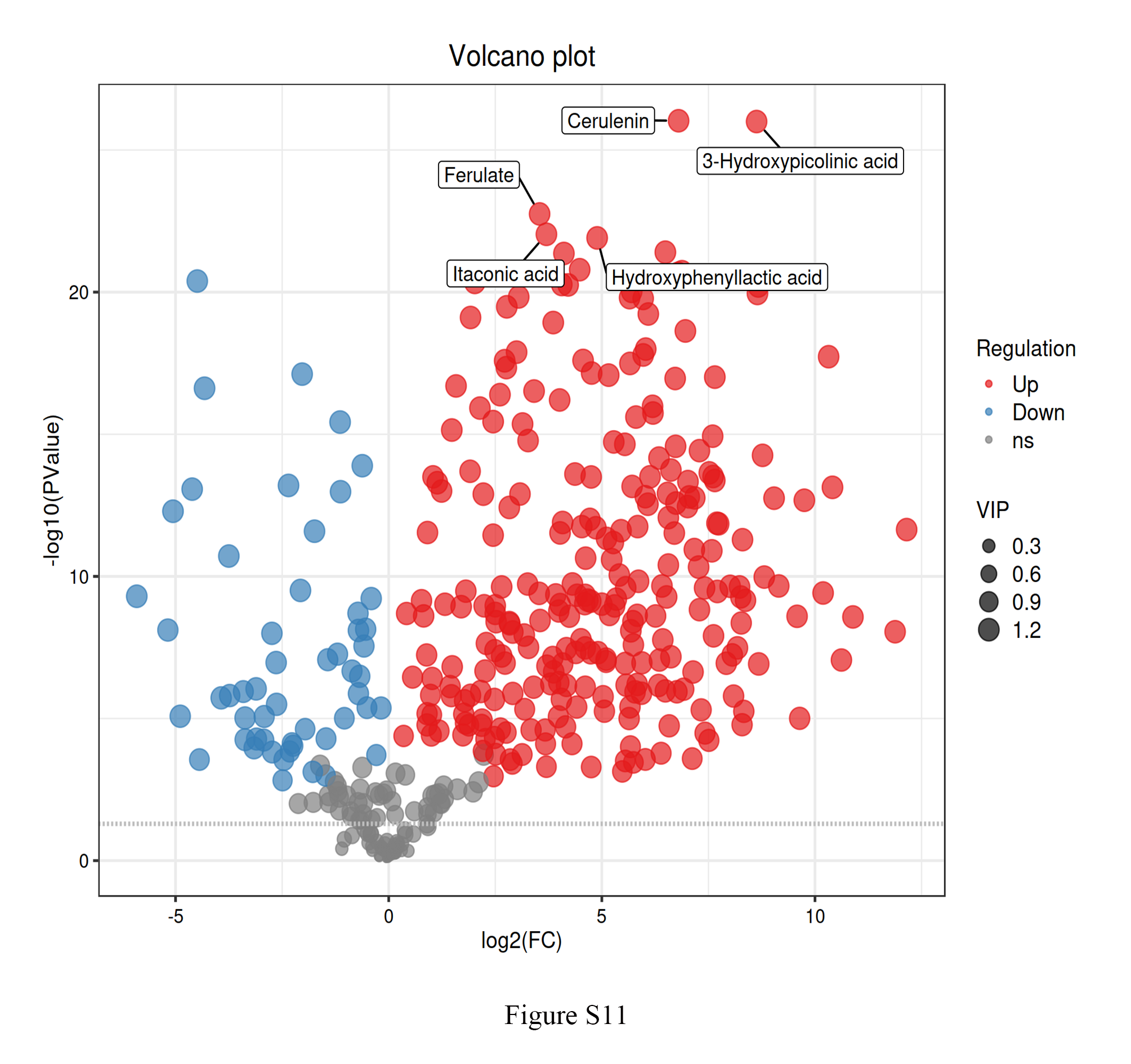

Supplement: Supplementary file 1 [file foods-14-03557-s001.zip › Figure S11 volcano redseed.tif]

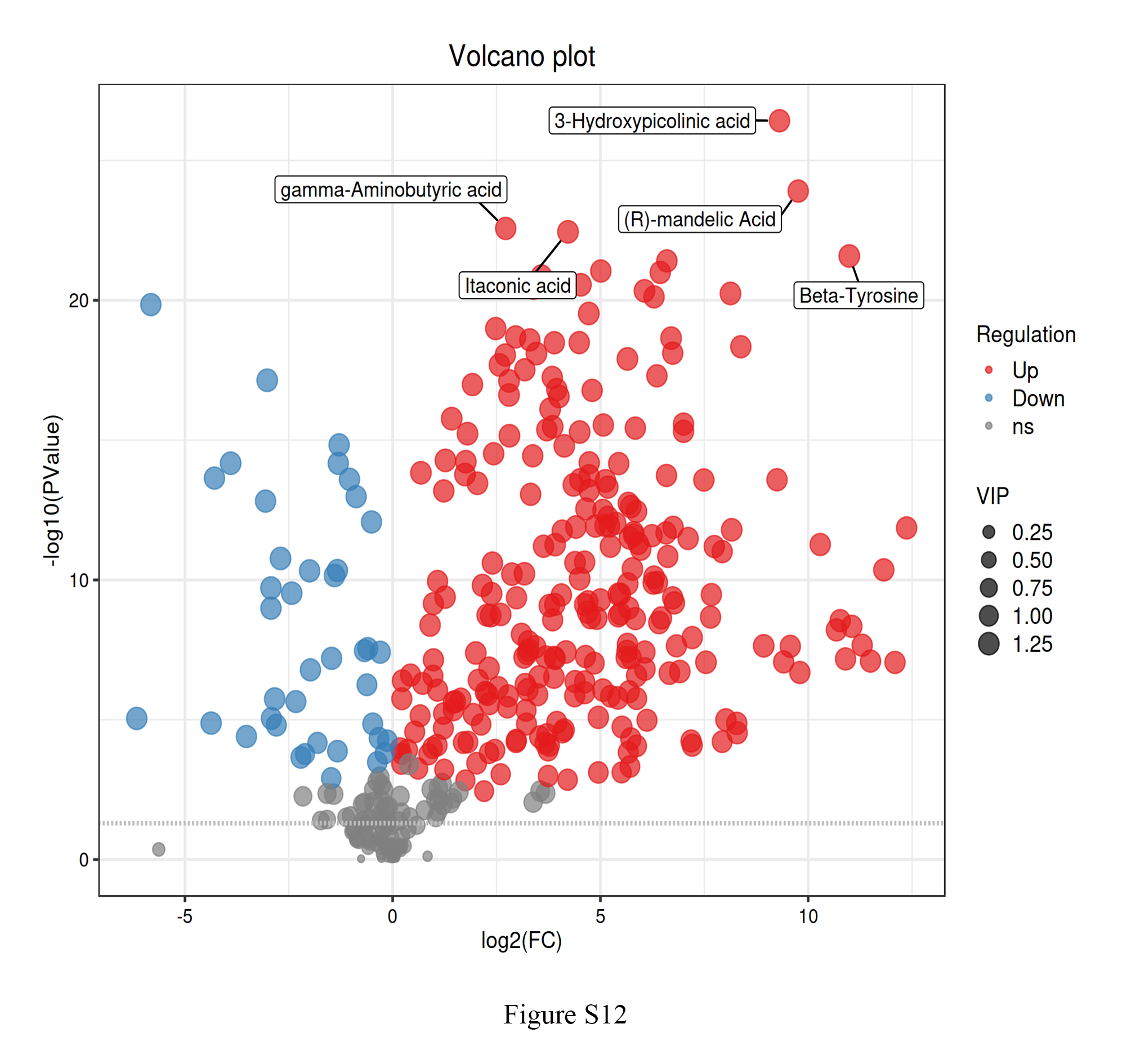

Supplement: Supplementary file 1 [file foods-14-03557-s001.zip › Figure S12 volcano blackseed.tif]

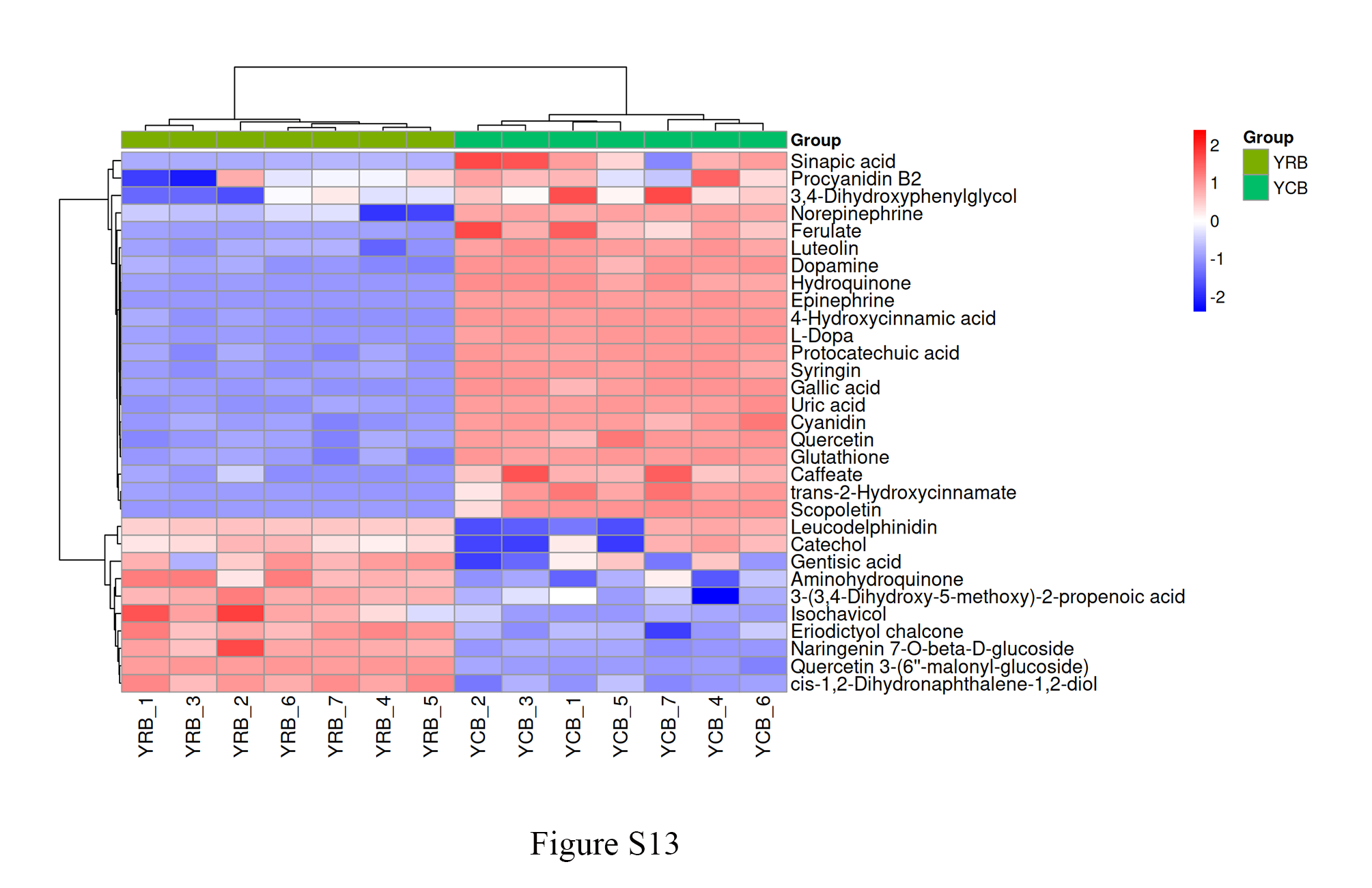

Supplement: Supplementary file 1 [file foods-14-03557-s001.zip › Figure S13 heatmap yellow.tif]

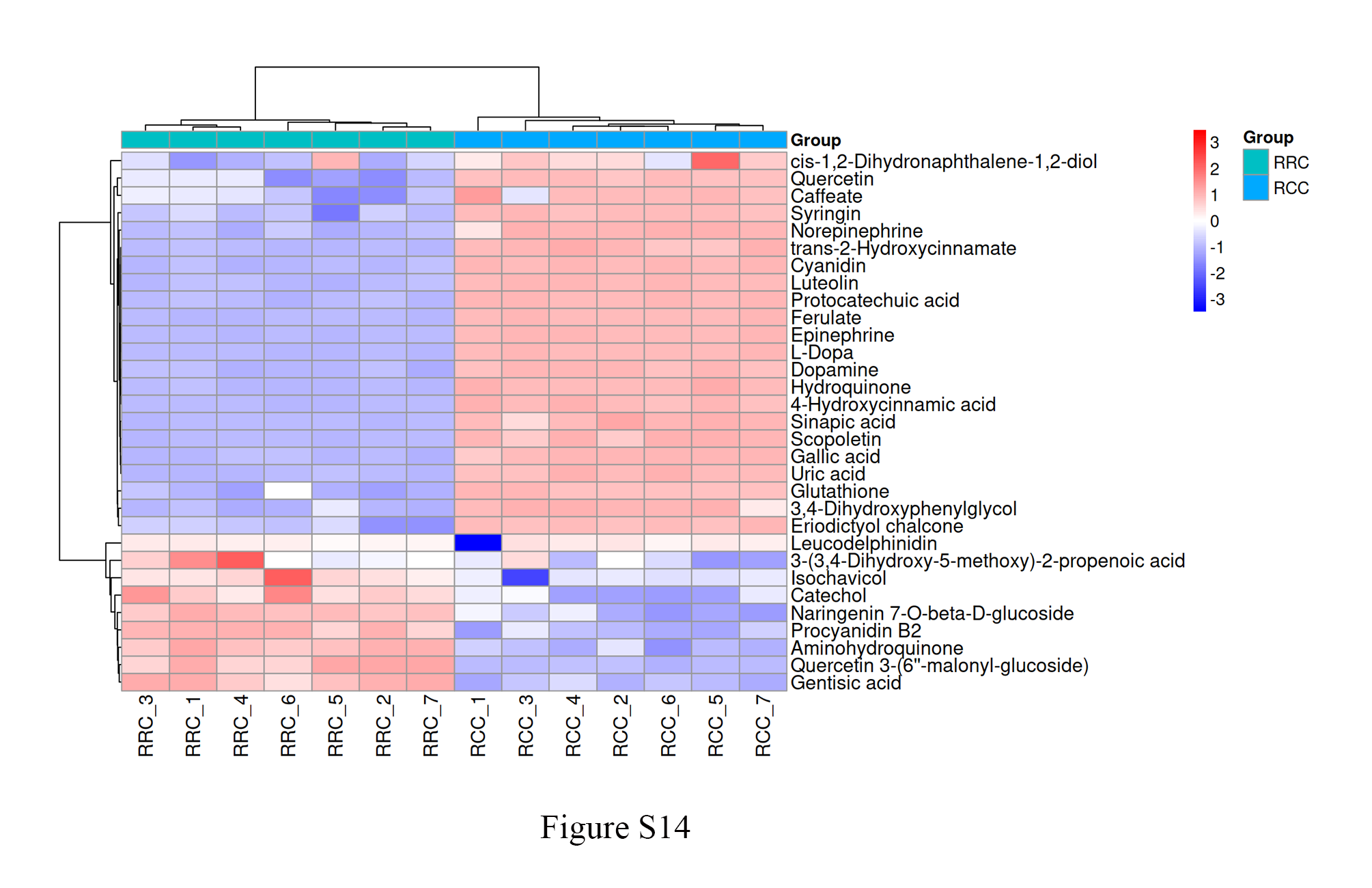

Supplement: Supplementary file 1 [file foods-14-03557-s001.zip › Figure S14 heatmap red.tif]

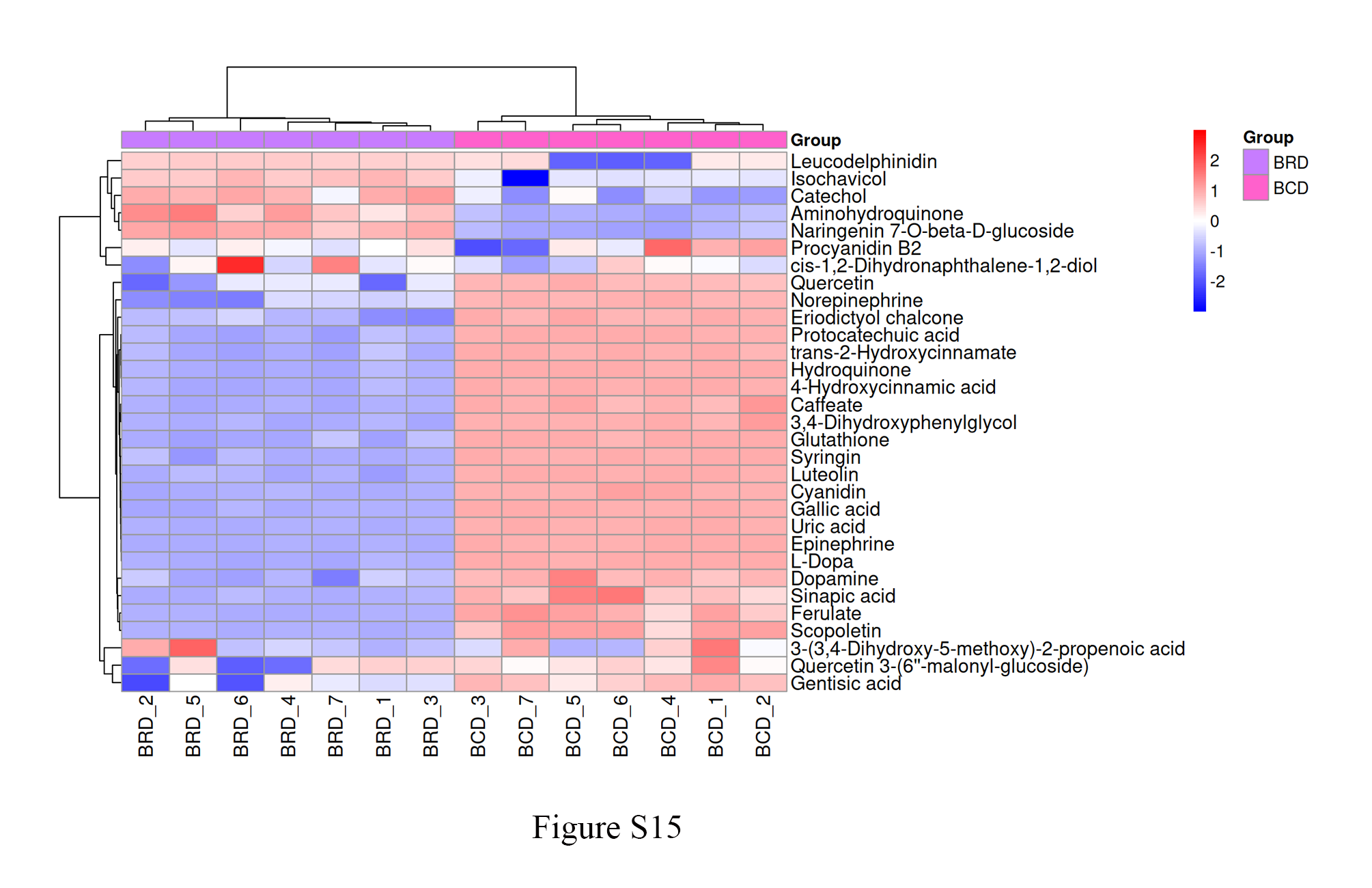

Supplement: Supplementary file 1 [file foods-14-03557-s001.zip › Figure S15 heatmap black.tif]

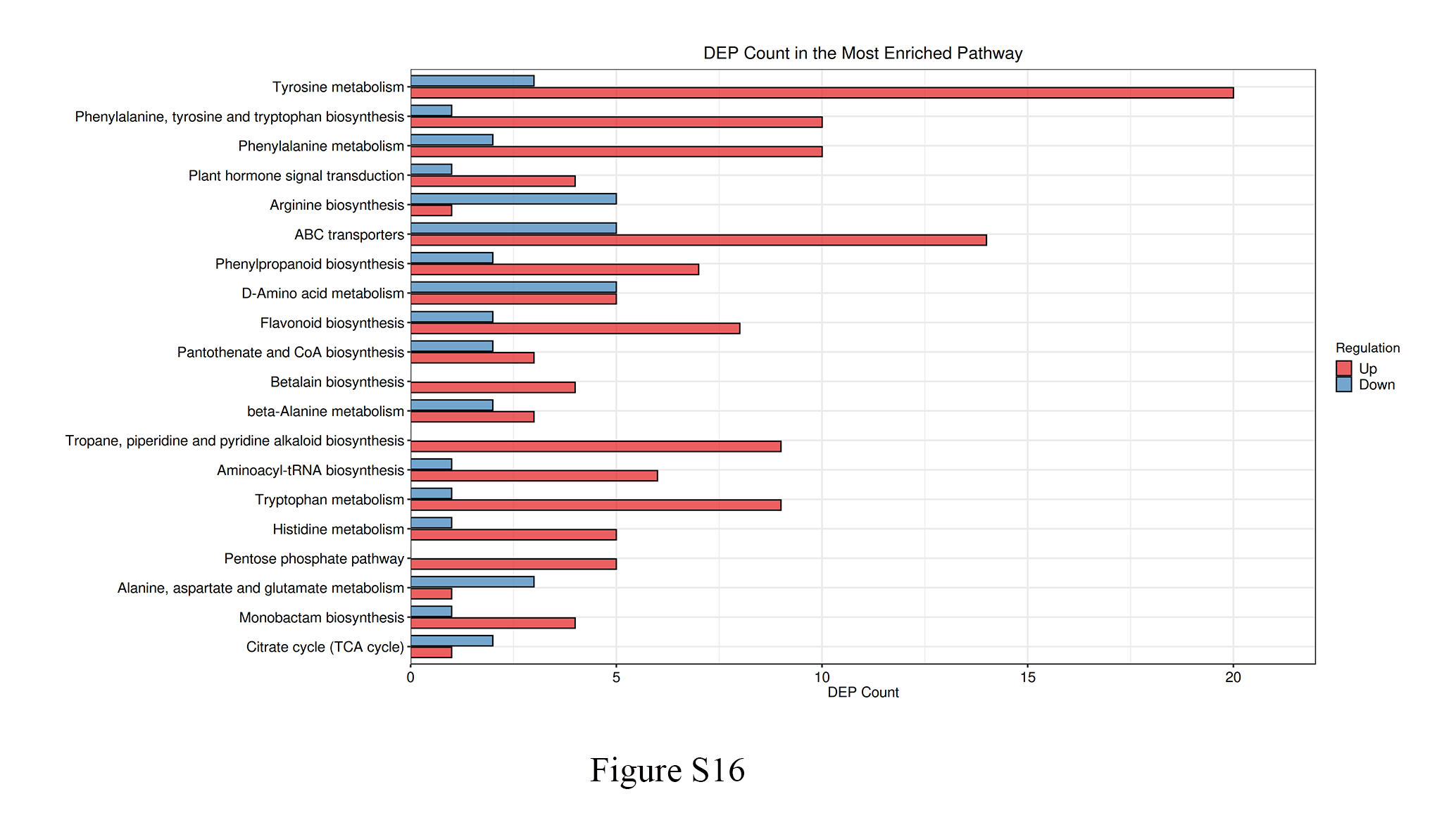

Supplement: Supplementary file 1 [file foods-14-03557-s001.zip › Figure S16 KEGGpathway yellow.tif]

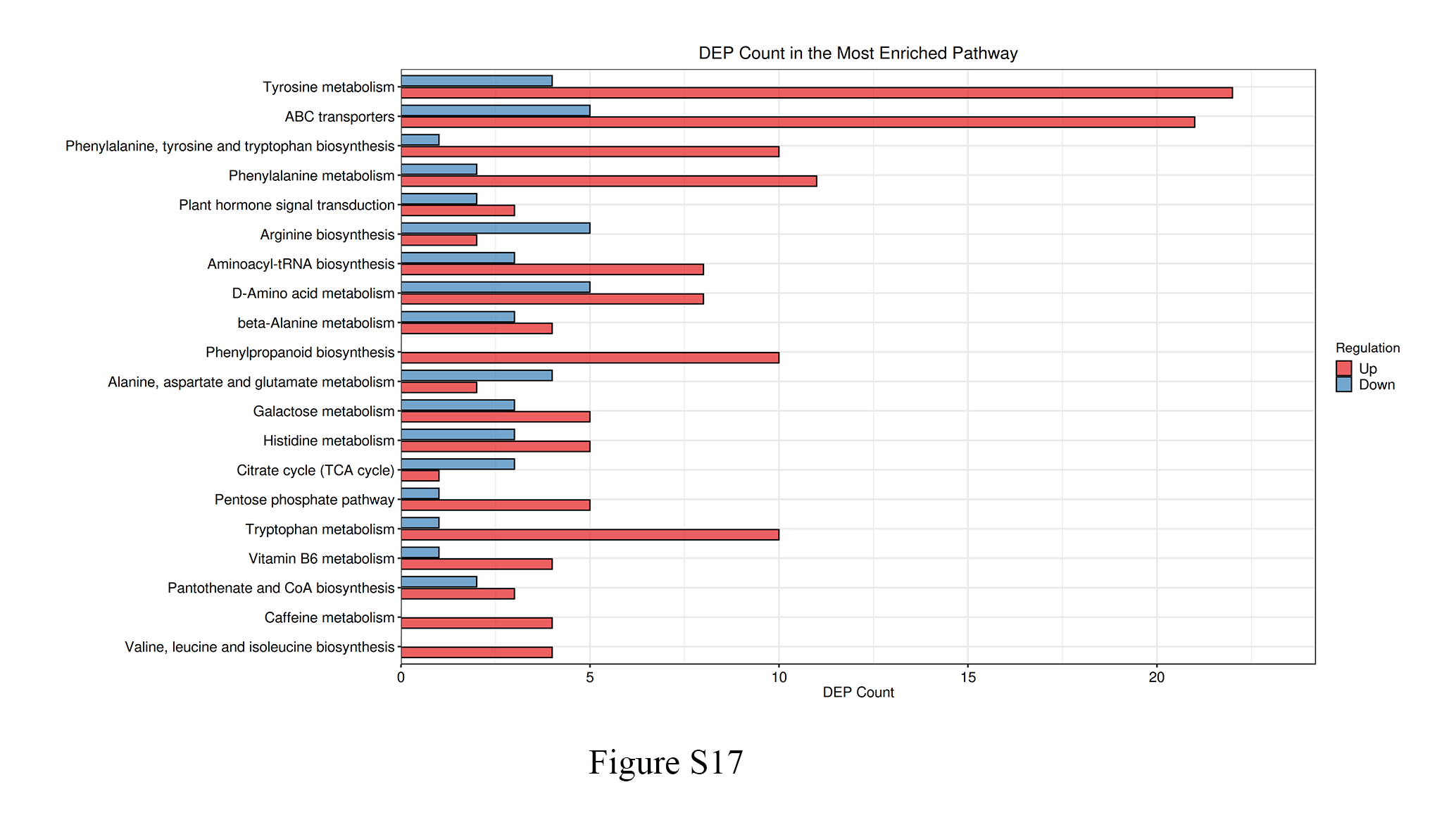

Supplement: Supplementary file 1 [file foods-14-03557-s001.zip › Figure S17 KEGGpathway red.tif]

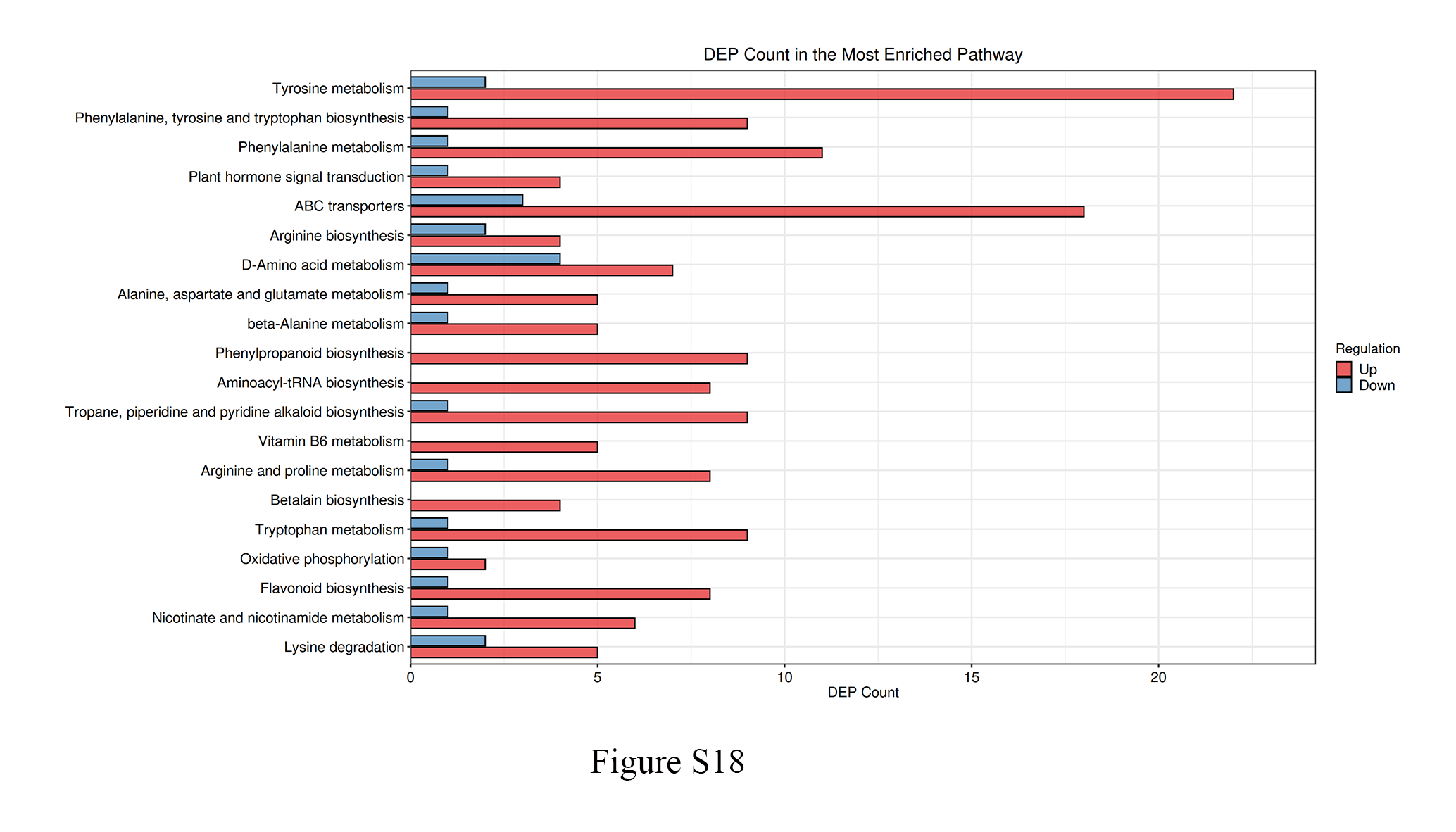

Supplement: Supplementary file 1 [file foods-14-03557-s001.zip › Figure S18 KEGGpathway black.tif]

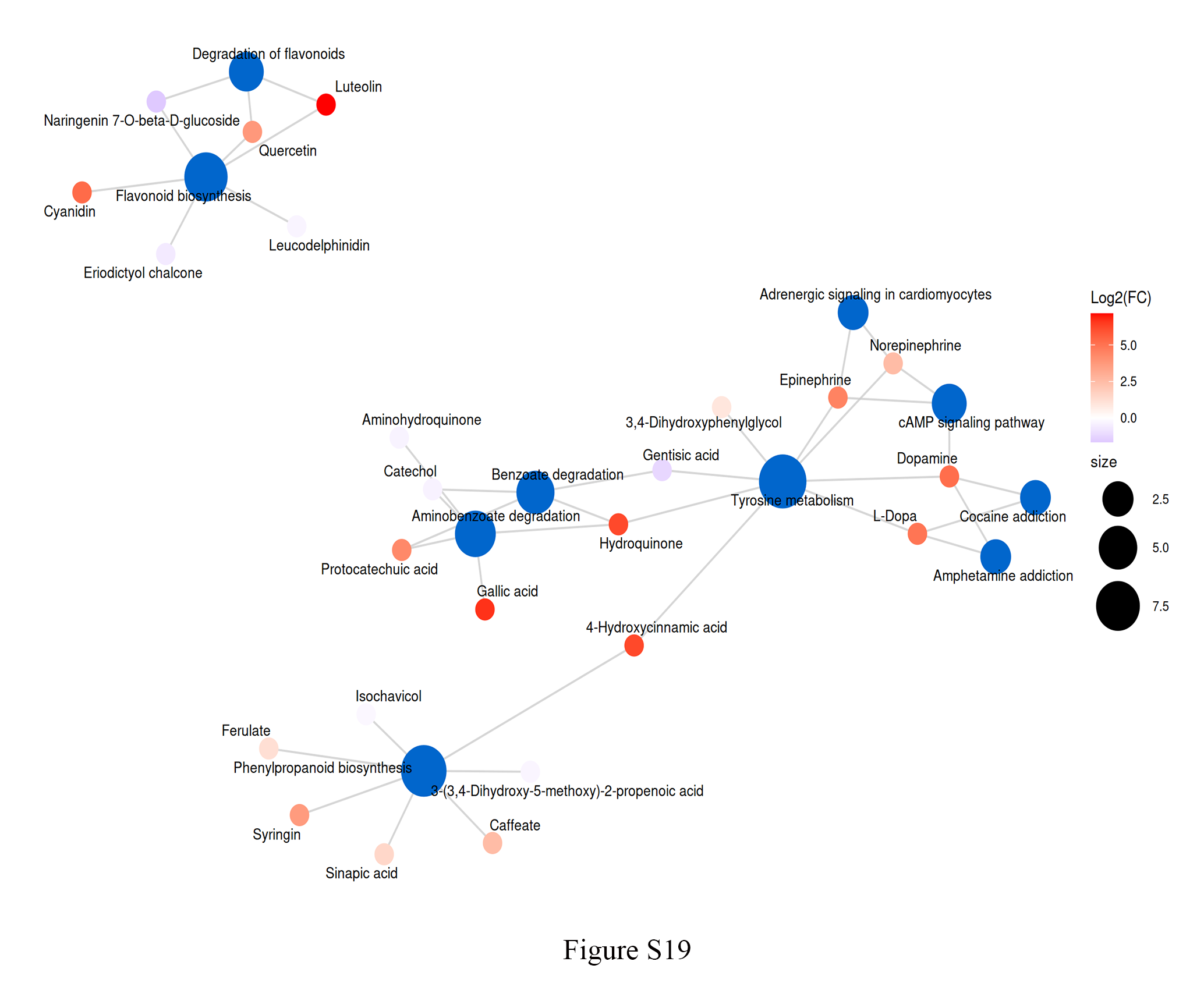

Supplement: Supplementary file 1 [file foods-14-03557-s001.zip › Figure S19 network yellow.tif]

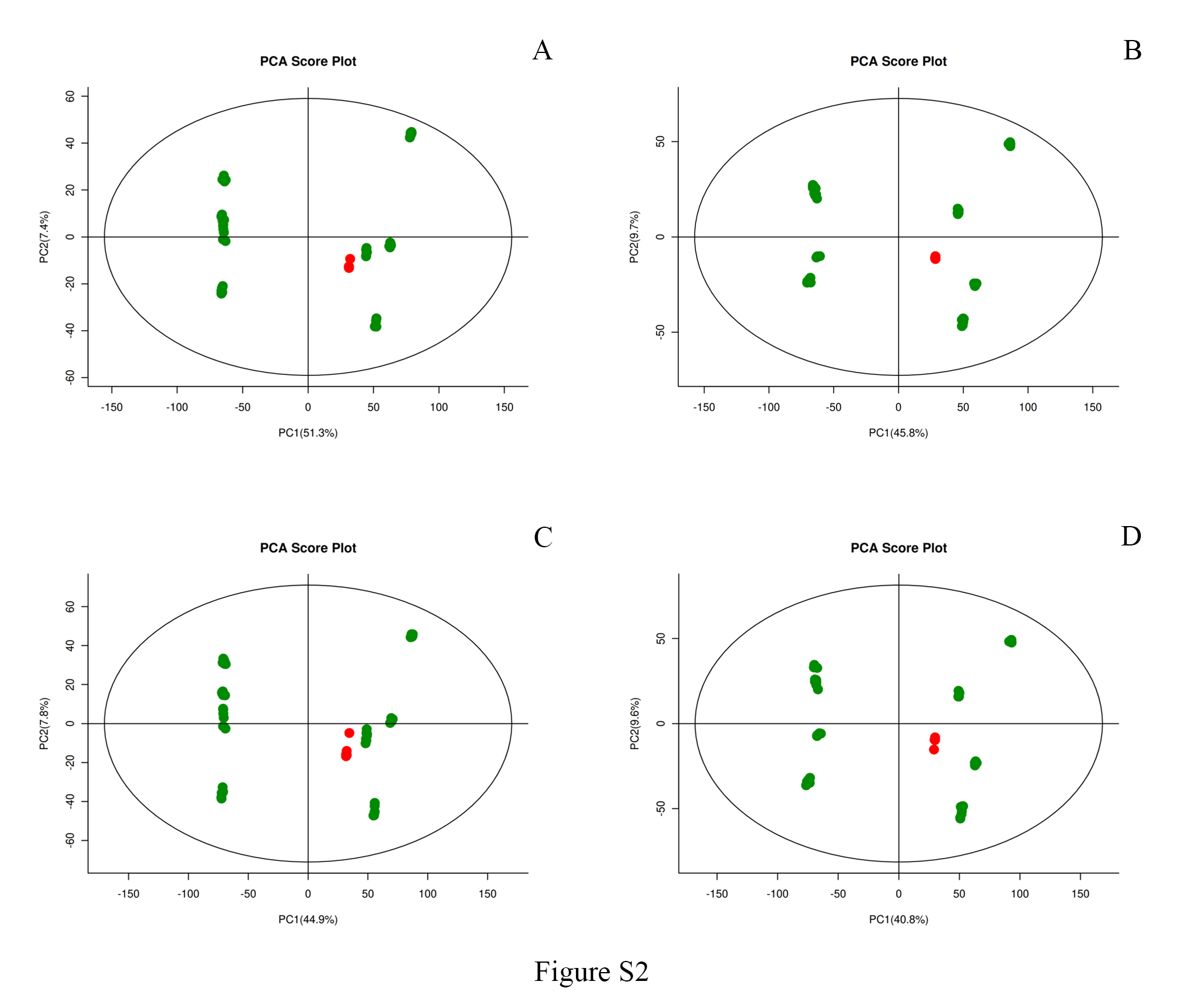

Supplement: Supplementary file 1 [file foods-14-03557-s001.zip › Figure S2 QA-QC.tif]

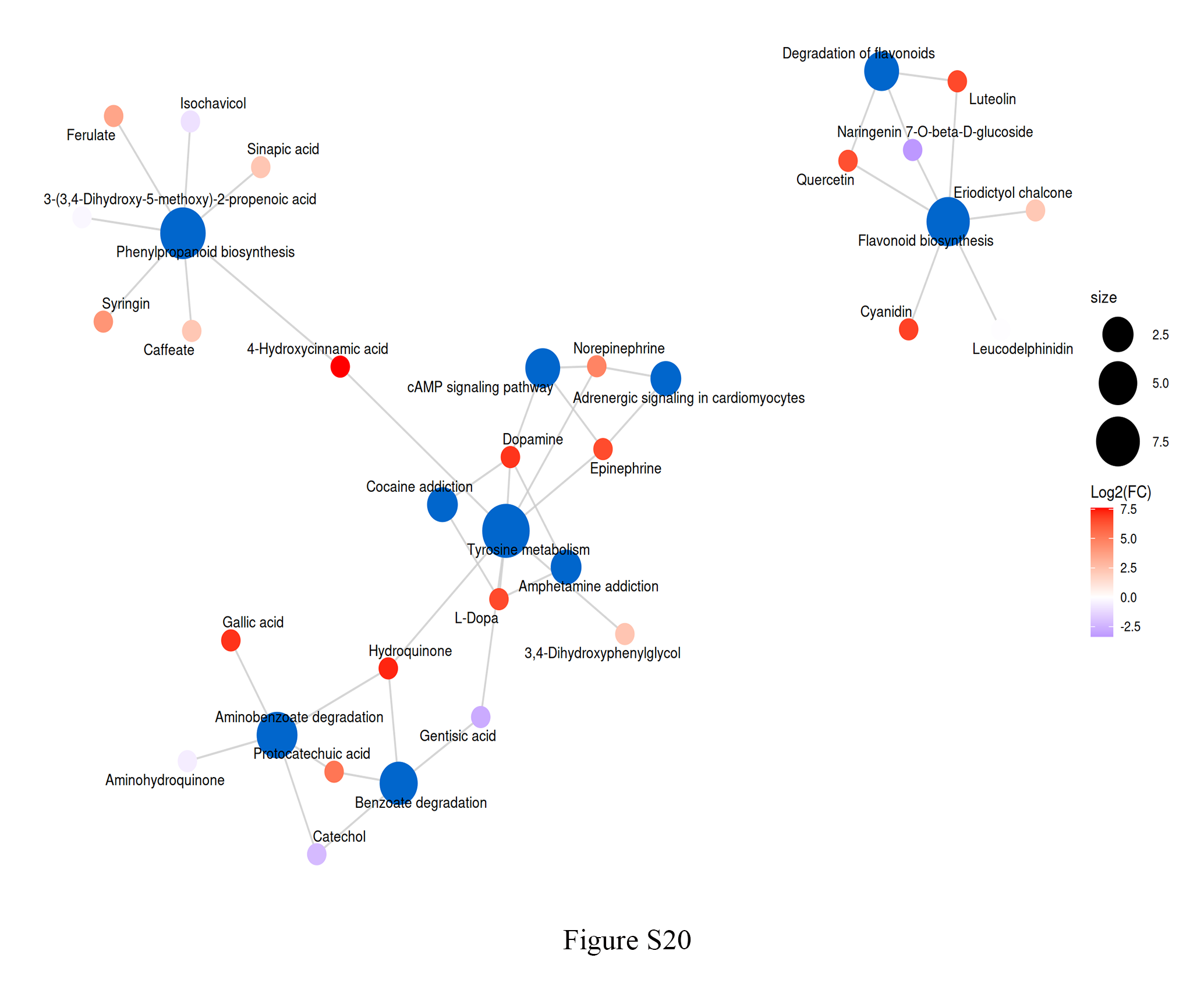

Supplement: Supplementary file 1 [file foods-14-03557-s001.zip › Figure S20 network red.tif]

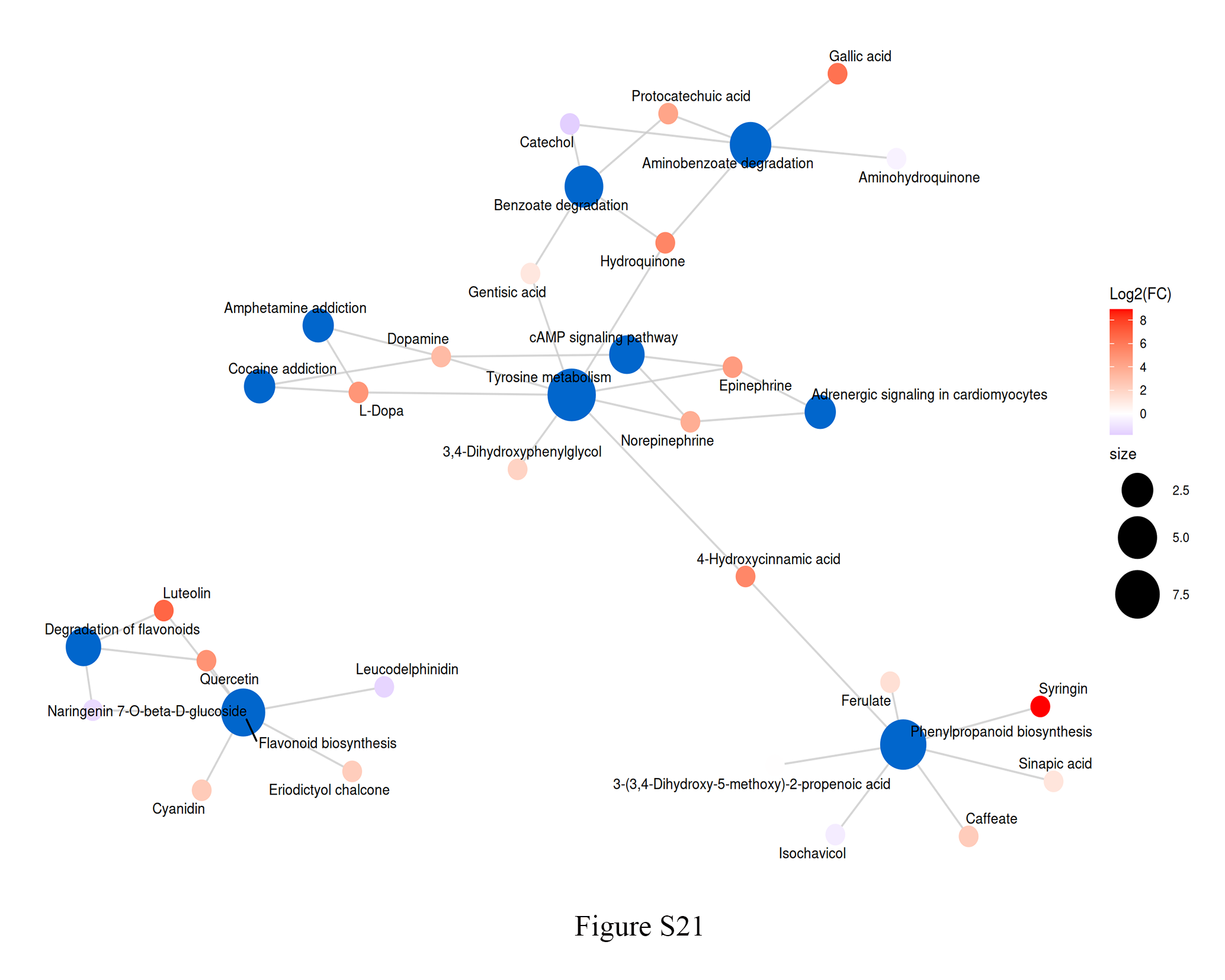

Supplement: Supplementary file 1 [file foods-14-03557-s001.zip › Figure S21 network black.tif]

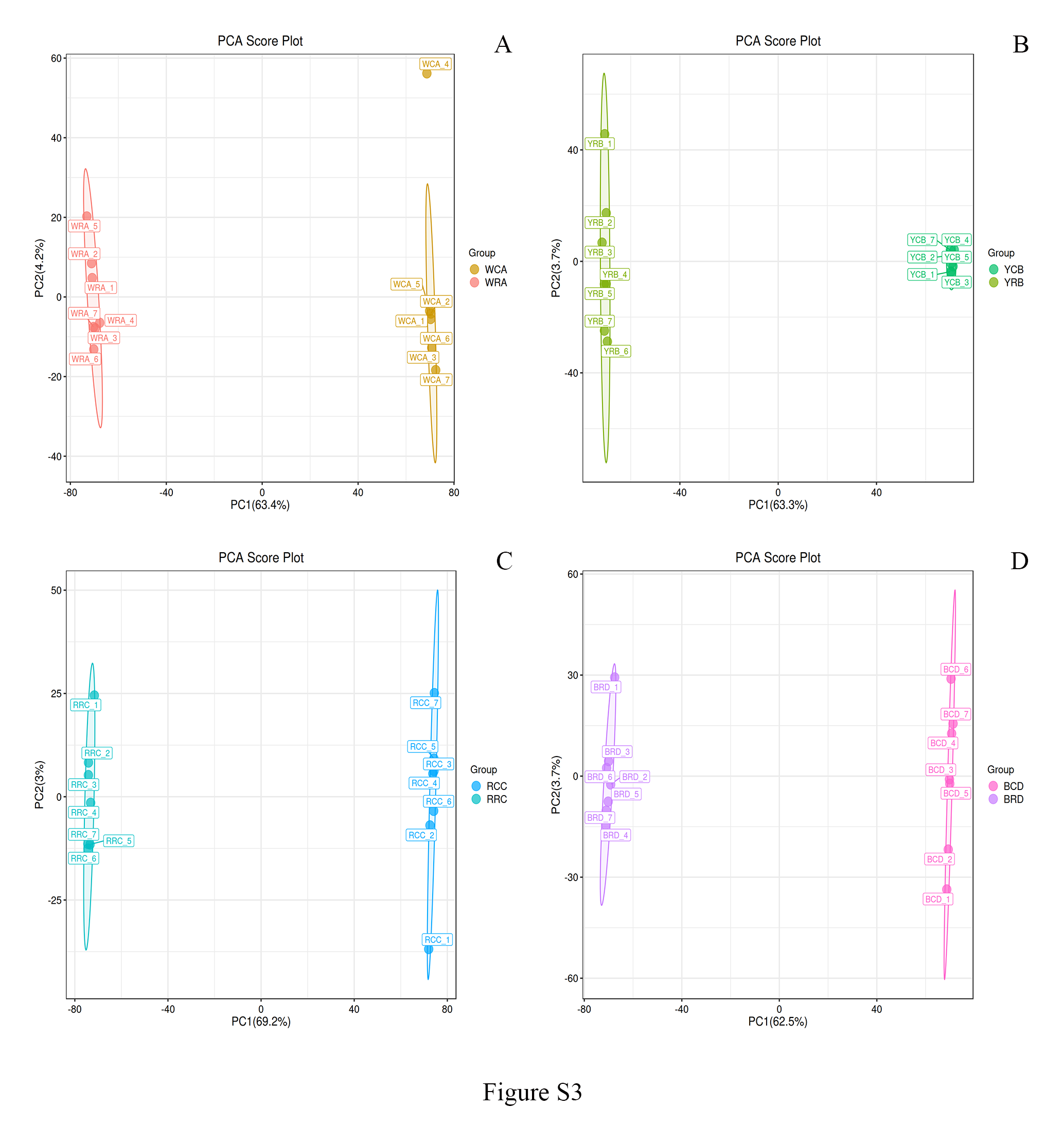

Supplement: Supplementary file 1 [file foods-14-03557-s001.zip › Figure S3 PCA nagative ion.tif]

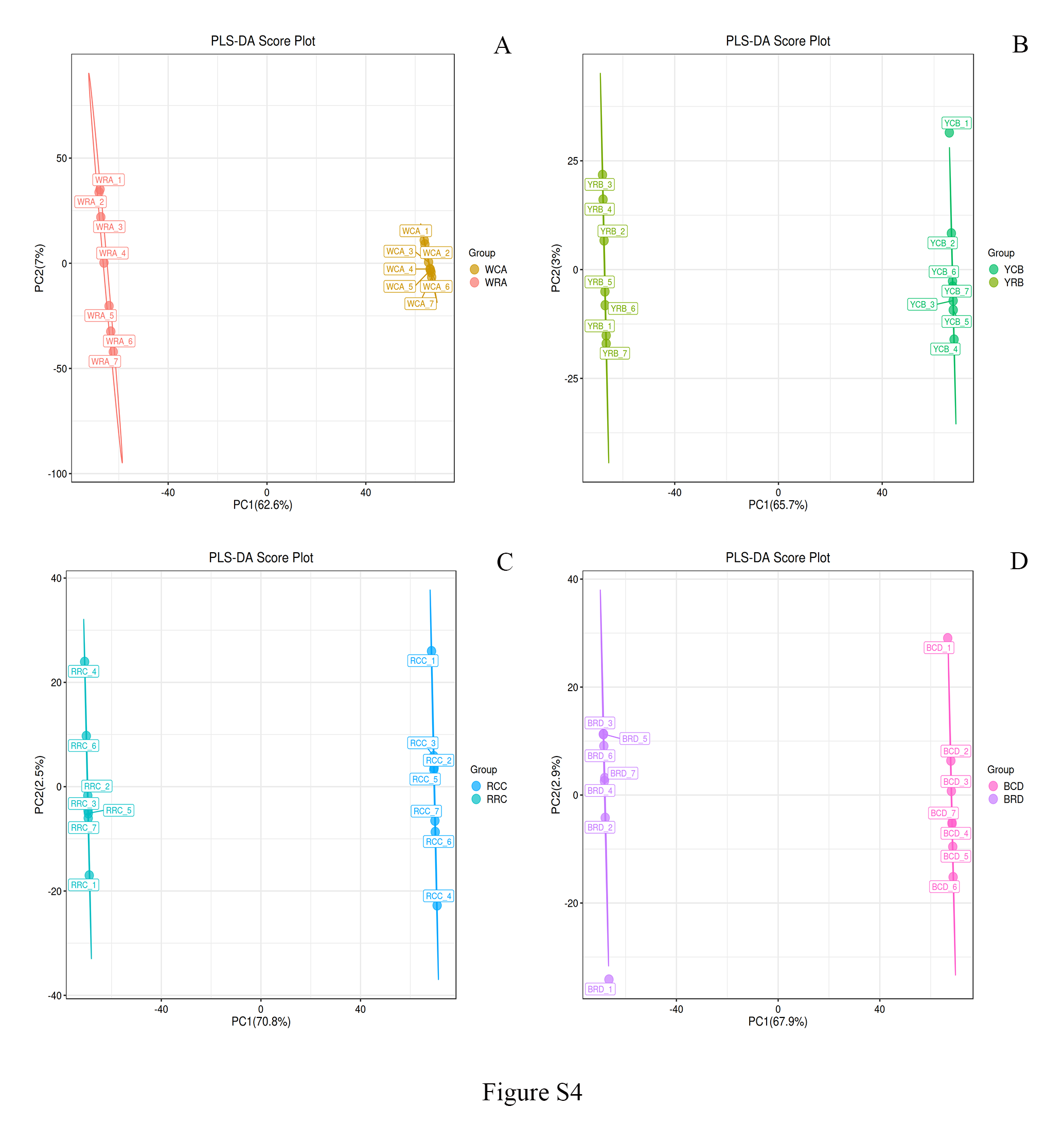

Supplement: Supplementary file 1 [file foods-14-03557-s001.zip › Figure S4 PLS-DA positive ion.tif]

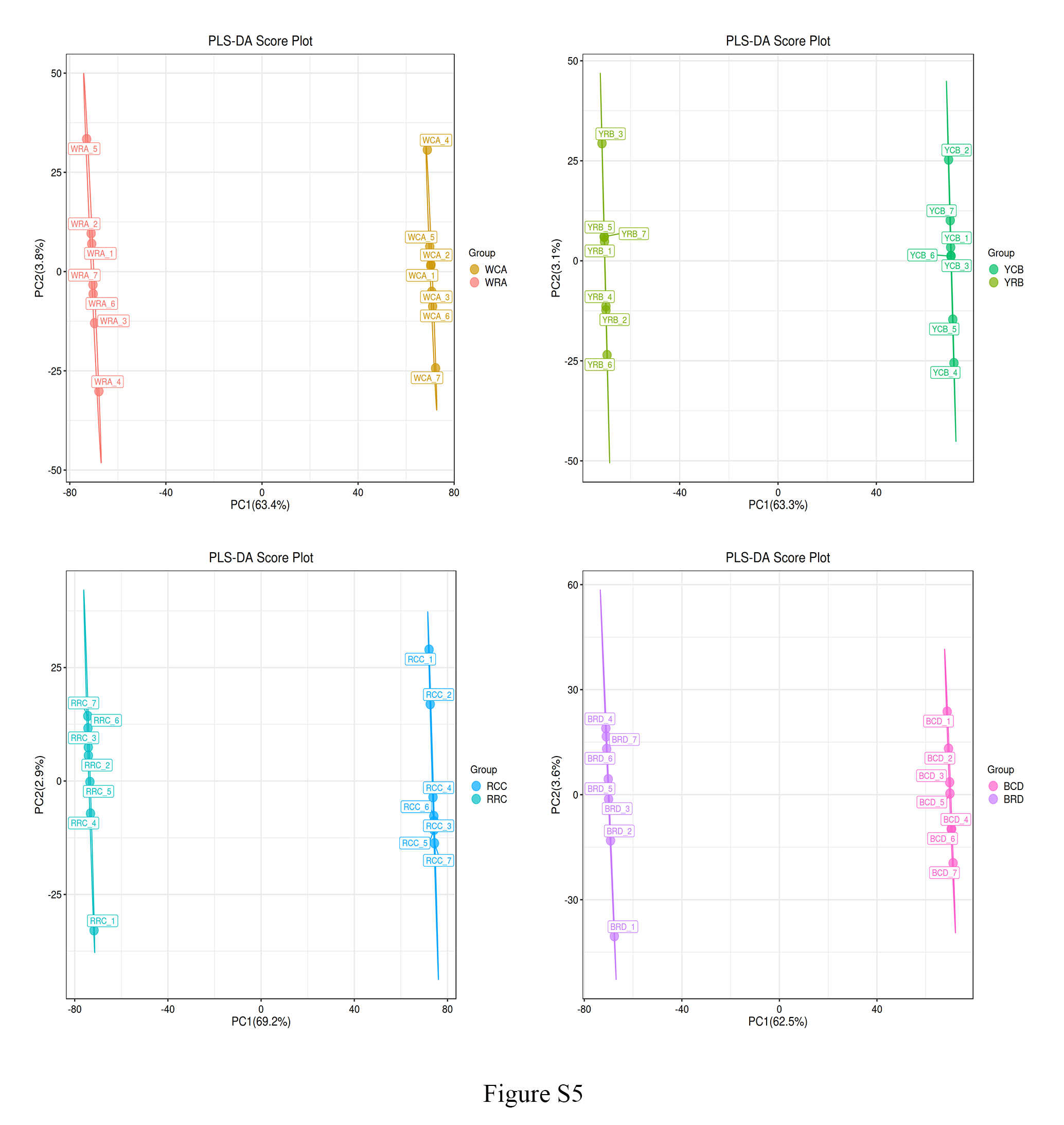

Supplement: Supplementary file 1 [file foods-14-03557-s001.zip › Figure S5 PLS-DA negative ion.tif]

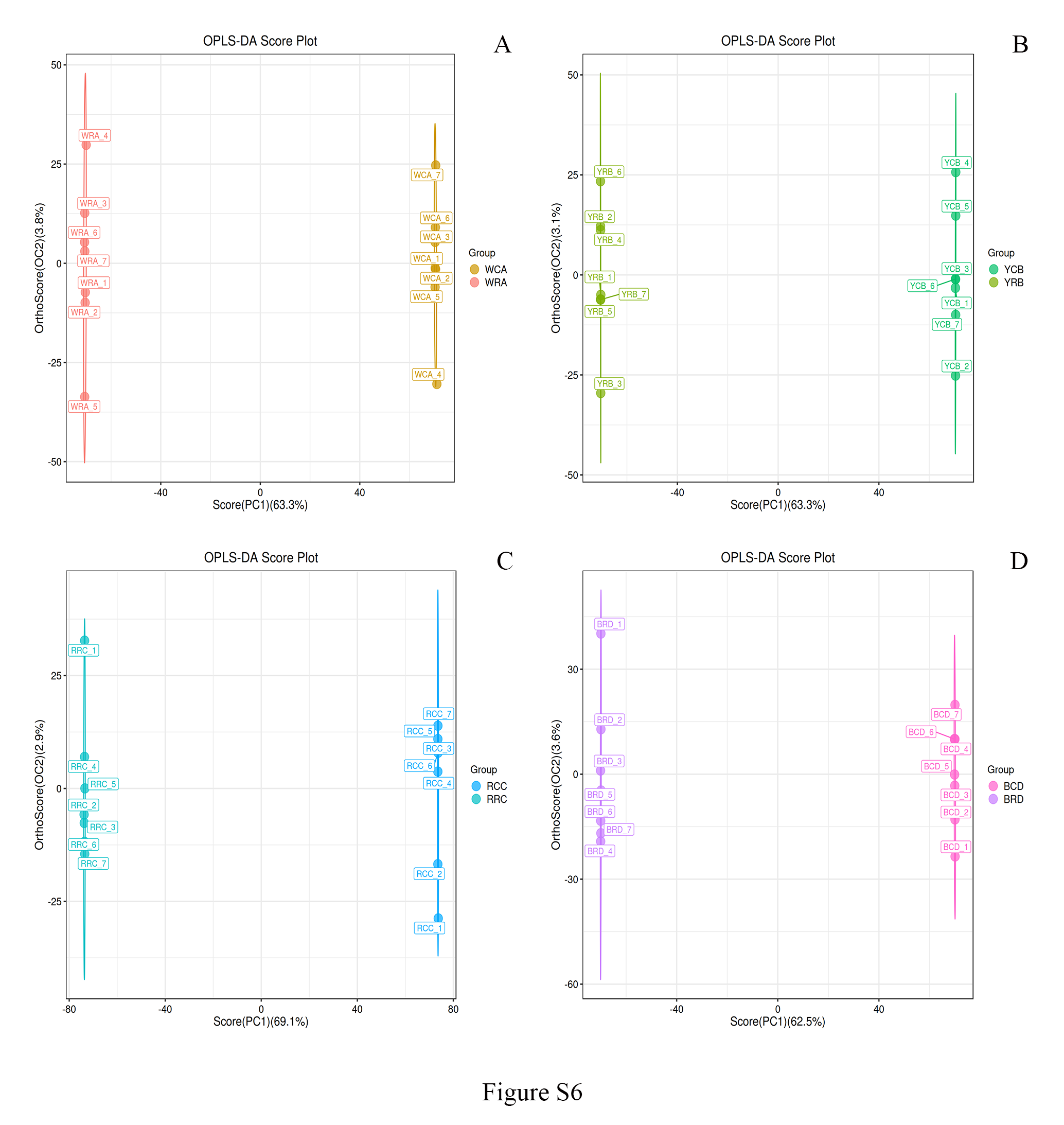

Supplement: Supplementary file 1 [file foods-14-03557-s001.zip › Figure S6 OPLS-DA scoreplot negative.tif]

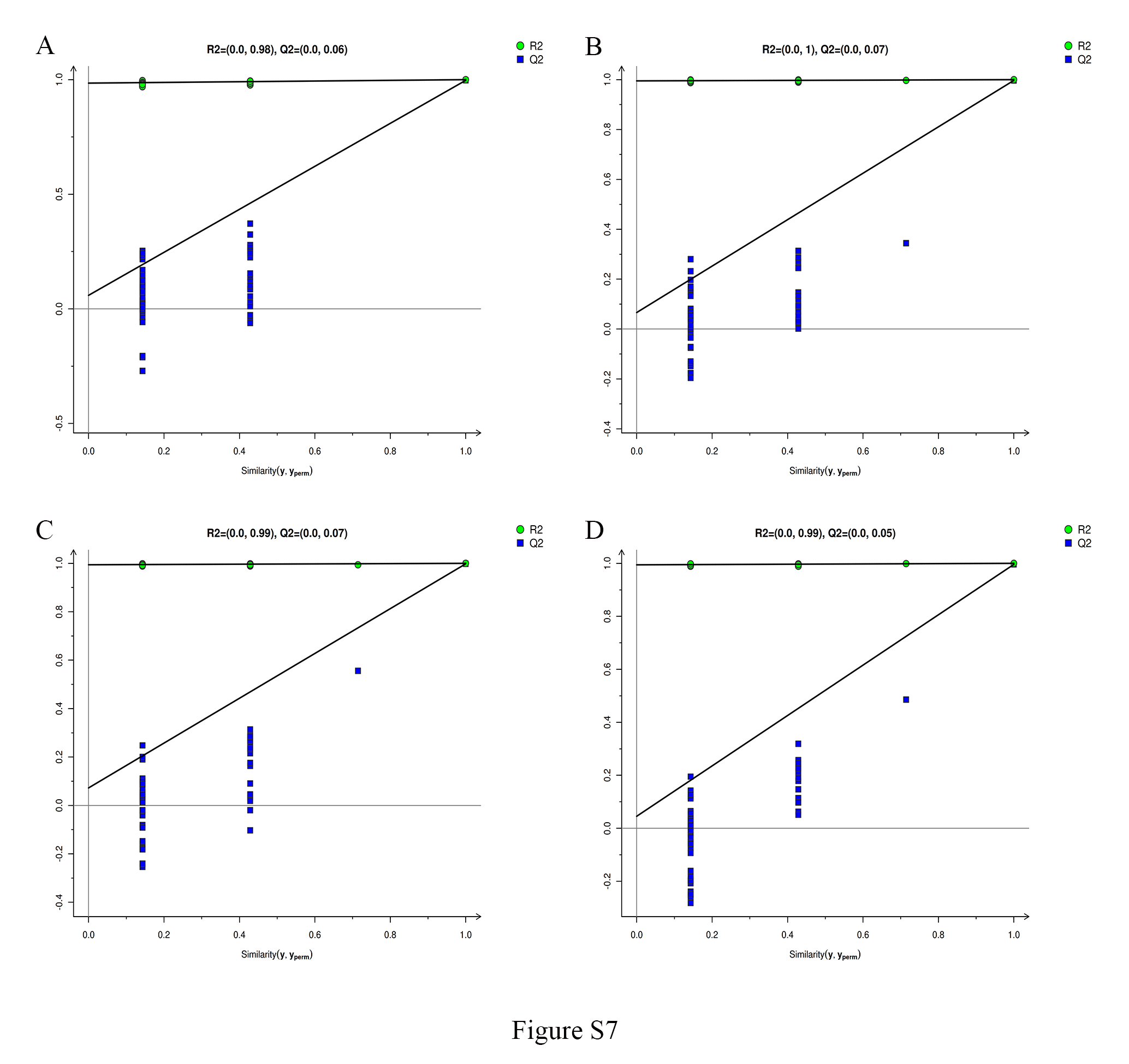

Supplement: Supplementary file 1 [file foods-14-03557-s001.zip › Figure S7 OPLS-DA displacement negative.tif]

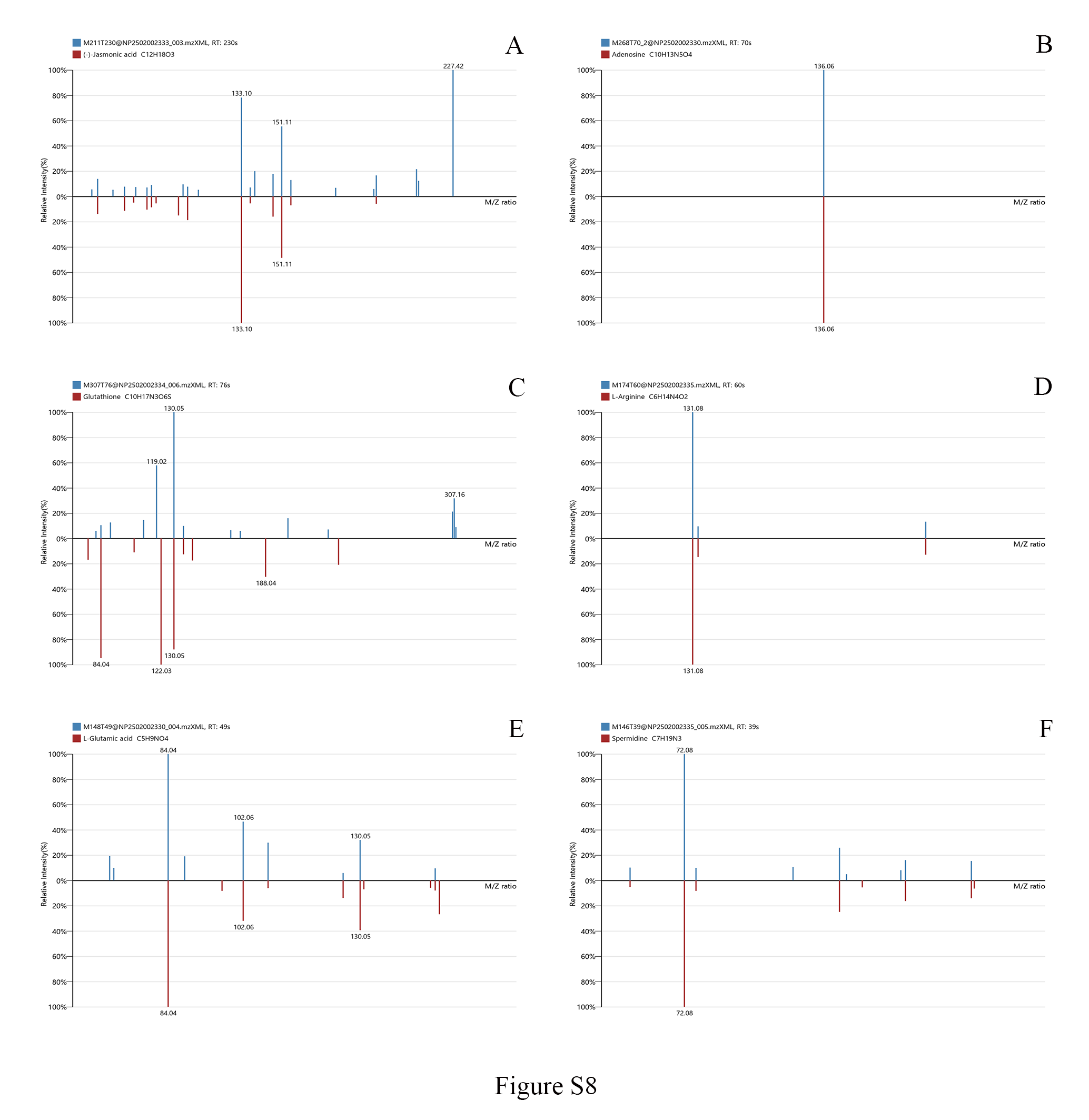

Supplement: Supplementary file 1 [file foods-14-03557-s001.zip › Figure S8 identification.tif]

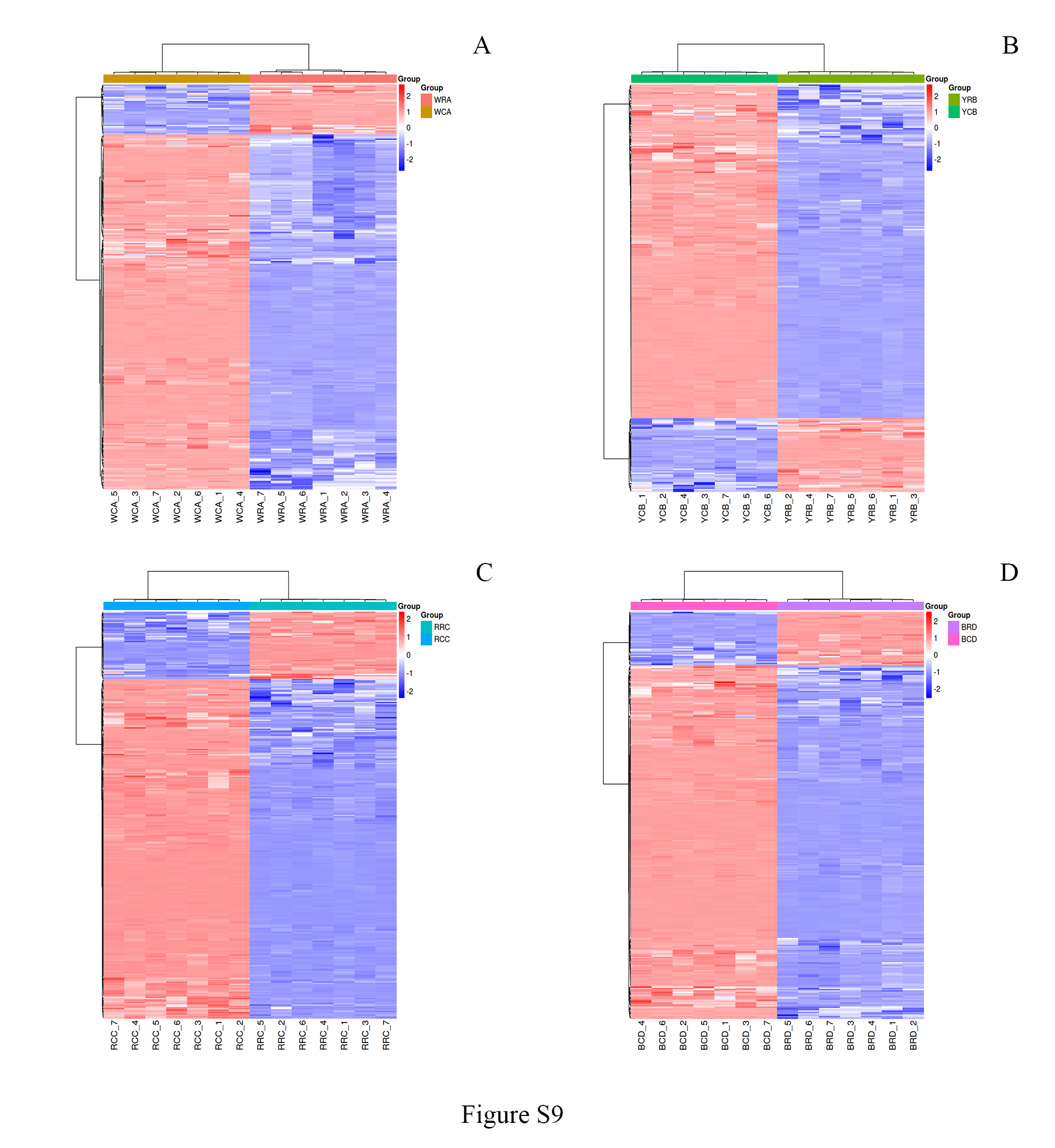

Supplement: Supplementary file 1 [file foods-14-03557-s001.zip › Figure S9 primary heatmap.tif]
